# Molecular characterisation of *Mycobacterium avium subsp. paratuberculosis* in Australia

**DOI:** 10.1186/s12866-021-02140-2

**Published:** 2021-04-01

**Authors:** Rachel Hodgeman, Rachel Mann, Keith Savin, Noel Djitro, Simone Rochfort, Brendan Rodoni

**Affiliations:** 1 Agriculture Victoria, AgriBio, La Trobe University, Bundoora, Victoria Australia; 2 School of Applied Systems Biology, AgriBio, La Trobe University, Bundoora, Victoria Australia

**Keywords:** *Mycobacterium avium subsp. paratuberculosis*, Johne’s disease, Phylogenetics, Single nucleotide polymorphism, Average nucleotide identity, IS*900*, IS*1311*

## Abstract

**Background:**

*Mycobacterium avium subsp. paratuberculosis* (Map) causes Johne’s disease (JD), a chronic enteritis widespread in ruminants, resulting in substantial economic losses, especially to the dairy industry. Understanding the genetic diversity of Map in Australia will assist epidemiological studies for tracking disease transmission and identify subtype characteristics for use in development of improved diagnostic typing methods. Here we investigated the phylogenetic relationships of 351 Map isolates and compared different subtyping methods to assess their suitability for use in diagnostics and accuracy.

**Results:**

SNP-based phylogenetic analysis of 228 Australian isolates and 123 publicly available international isolates grouped Type S and Type C strains into two distinct lineages. Type C strains were highly monomorphic with only 20 SNP differences separating them. Type S strains, when aligned separately to the Telford strain, fell into two distinct clades: The first clade contained seven international isolates while the second clade contained one international isolate from Scotland and all 59 Australian isolates. The Australian Type B strain clustered with US bison strains. IS*1311* PCR and Restriction Enzyme Analysis (REA) intermittently generated incorrect results when compared to Long Sequence Polymorphism (LSP) analysis, whole genome SNP-based phylogenetic analysis, IS*1311* sequence alignment and average nucleotide identity (ANI). These alternative methods generated consistent Map typing results. A published SNP based assay for genotyping Map was found to be unsuitable for differentiating between Australian and international strain types of Map.

**Conclusion:**

This is the first phylogenetic analysis of Australian Map isolates. The Type C lineage was highly monomorphic, and the Type S lineage clustered all Australian isolates into one clade with a single Scottish sheep strain. The Australian isolate classified as Type B by IS*1311* PCR and REA is likely to be descended from bison and most closely related to US bison strains. Limitations of the current typing methods were identified in this study.

**Supplementary Information:**

The online version contains supplementary material available at 10.1186/s12866-021-02140-2.

## Background

*Mycobacterium avium subsp. paratuberculosis* (Map) is a subspecies of the *Mycobacterium avium* complex (Mac) [1] and is the causative agent of Johne’s disease, a chronic enteritis in ruminants, predominately cattle and sheep. Johne’s disease in dairy cattle is widespread globally and is characterised by reduced fertility and milk production followed by chronic and progressive loss of body condition, intermittent or persistent diarrhoea [2] and, in most chronic cases, death. In sheep and other small ruminants, clinical signs of disease include chronic weight loss, occasionally oedema and in more advanced cases, hypoalbuminemia [3]. Infected animals cause substantial economic losses to the farming industry. In 2016, it was estimated that dairy farms in Australia suffered an annual loss of AUS$11,748 per farm [4] and an average loss of AUS$64,100 per sheep farm [5].

The main route of transmission of Map is by the faecal-oral route through the ingestion of milk, water, colostrum or feed that has been contaminated by Map in faeces [6]. Of concern is the association of Map with Crohn’s disease in humans. Since the first discovery of Crohn’s disease there has been no established etiological agent, however the same disease pathology has been shown in Johne’s disease in ruminants as has been seen in patients with Crohn’s disease [7]. Map has been found in milk and other dairy products [8] suggesting a route of transmission to humans and has been isolated from intestinal tissue, blood and breast milk of Crohn’s disease patients [9–12].

Strain typing of bacterial pathogens is important for studying the phylogeny, epidemiology and population structure amongst isolates [13]. It can also identify the genetics influencing phenotypic characteristics of pathogens such as antibiotic resistance, host specificity, virulence, and pathogenicity [14]. Strain typing can be very challenging for Map as it is genetically monomorphic [14]. Two strain types of Map were first identified through their growth characteristics, pathogenicity and host preference: Type I (Sheep (S) type) and Type II (Cattle (C) type) [15]. With advances in molecular typing techniques, a new strain type was identified by pulse field gel electrophoresis, Type III (a subtype of S) [16] and the identification of small sequence polymorphisms in the Map genomic sequence identified a bison type, Type B [17]. The most common method for differentiating Map strains is by IS*1311* genotyping which relies on detecting the number of copies of the IS*1311* insertion sequence with a C or T at base position 223: Type B has a T at position 223 in all copies, Type S has a C at position 223 in all copies, and Type C has one or more copies with a C or T at position 223 [18]. However, some studies have shown that this method does not accurately differentiate between Type C strains and Type S strains [19].

Whole genome sequencing of Map has identified genomic deletions, insertions and rearrangements within Map isolates which has allowed the identification of other target regions for strain differentiation such as the presence or absence of long sequence polymorphisms (LSPs) [20]. Twenty LSPs have been identified that are highly specific for Map [21] and a subset of three LSPs (LSP-4, LSP-18 and LSP-20) have been used for strain typing [22]. LSP-4 is a large sequence located within the mycobactin synthesis operon and only present in Type S strains. LSP-18 is a sequence that is present in Type S strains but is replaced by an insertion sequence, the IS*900* element, in the Type C strains [23]. LSP-20 encodes a protein involved in metabolism and has only been detected in Type C strains [23].

Map is genetically monomorphic [18] which has made genotyping very difficult using traditional methods. Improved technology and reduced costs of high throughput sequencing has enabled whole genome sequencing of large numbers of bacterial isolates for comparison in diagnostic settings. Whole genome comparisons can improve our understanding of Map subtypes, mechanisms of transmission and virulence, vaccine development and management practices for subtypes circulating within populations as well as support the development of advanced diagnostic tools. Single nucleotide polymorphisms (SNPs) are being used more and more as a typing and tracing tool, especially for monomorphic bacteria [13]. SNP typing has been used for differentiation and tracing of *M. tuberculosis* [24], *M. bovis* [25], *Salmonella enterica* serovar *Typhi* [26], *Yersinia pestis* [27], *Bacillus anthracis* [28] and *Escherichia coli* [29]. However, there have been limited studies done on SNP typing of Map [13]. One such SNP typing method for strain differentiation of Map is the identification of SNPs located in the *gyr*A and *gyr*B gene [30]. This typing scheme has been successful in differentiating type I, II and III of Map strains [31]. One of the advantages of SNP typing is that stable, informative loci can be selected from the core genome, avoiding mobile elements, for further analysis.

In this study SNP analysis of core genome sequences was used to determine the phylogenetic diversity of Australian Map isolates that have been collected over the last 35 years and stored in the Australian Johne’s Disease Reference Collection (AJDRC). This SNP analysis included international isolates to get a broader context of the diversity of Australian Map populations. A comparison of the PCR-based IS*900* and IS*1311* typing methods to whole genome sequencing-based analysis of LSP, SNP analysis, ANI and phylogenetic relationships was conducted to assess their suitability for differentiating Map strains.

## Results

### IS*900* PCR and IS*1311* PCR and REA of Australian map isolates

All 228 Australian isolates from the AJDRC examined in this study were mycobactin dependent and IS*900* PCR positive. Based on IS*1311* PCR and REA analysis there were 165 Type C isolates, 59 Type S isolates, two Type B isolates and four Mac isolates (additional file 1: Table S1). Cross species transmission (e.g. Type C identified from sheep and Type S identified from cattle) was identified to have occurred with eight Australian isolates, based on REA analysis conducted in this study (additional file 3: Table S2). *Mycobacterium avium subsp. avium* was isolated from three infected cattle, Map Type C was isolated from four infected sheep and Type S was isolated from one infected cow. These are historical isolates and no clinical information on infection status was recorded at the time of isolation.

### Genome sequence, assembly and average nucleotide identity of Australian and international map isolates

**Fig. 1:**
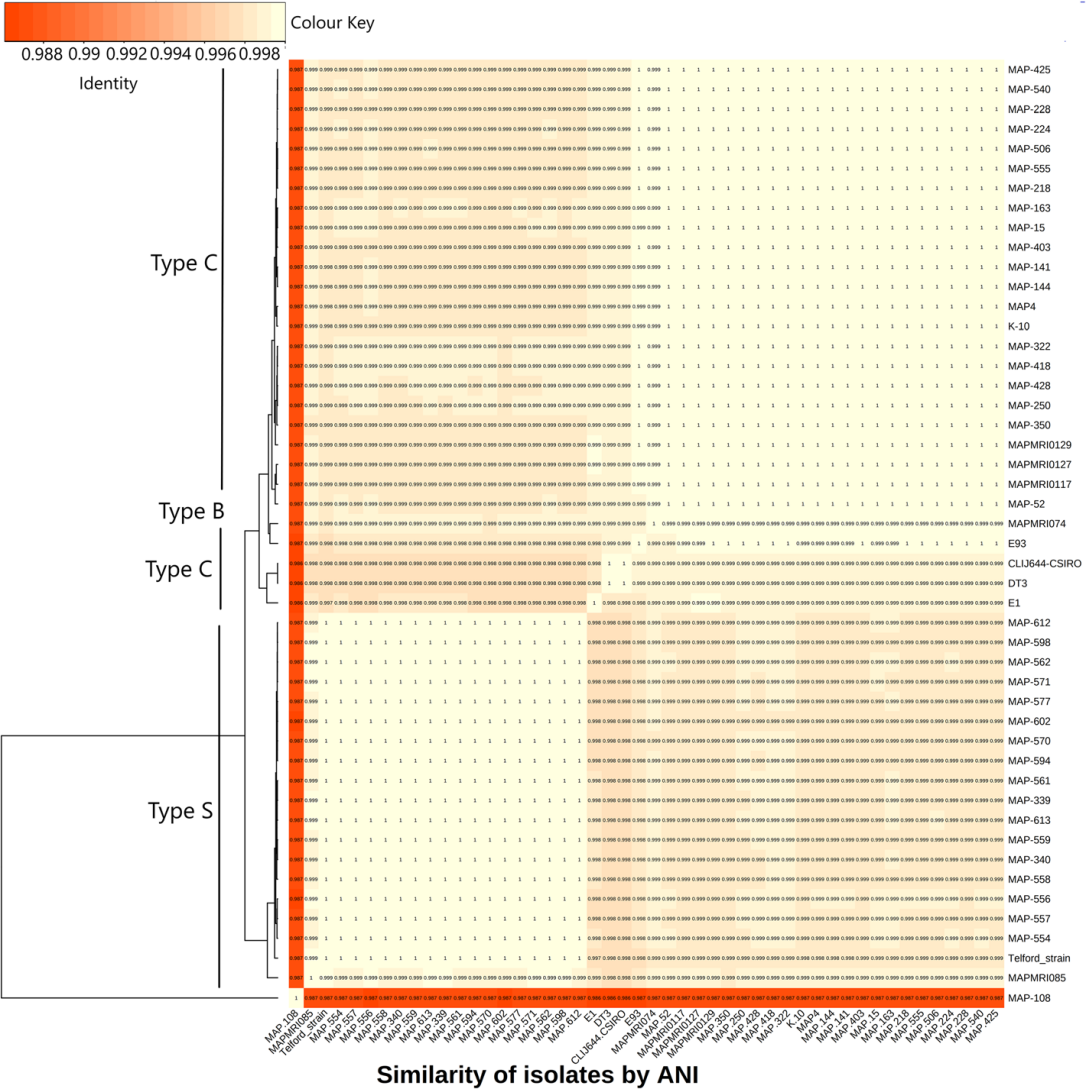
A heat map and dendrogram of average nucleotide identity (ANI) between 50 selected Australian Map isolates and international sequences. ANI values are depicted as a colour gradient with Yellow = 1 (100% ANI), orange = 0.87 (87% ANI)

Whole genome sequence data was generated for 230 Australian isolates. The genome length of all but one Australian Map isolates was 4.7 Mb with a GC content of 69%. One Australian isolate, MAP-116, had a total length of 5.6 Mb and a GC content of 67%, indicating that this isolate was not Map. Average depth of coverage, number of raw reads, N50 and number of SNPs for each isolate is presented in additional file 4, Table S3. ANI was calculated between pairs of 50 representative genome sequences of Australian and international isolates analysed in this study and is presented as a heat map in Fig. 1. There was a 0.02% difference between the ANIs of Type C and Type S strains. All Type C strains shared an ANI of 99.9%, all Type S strains shared an ANI of 99.8% and all Type B strains shared an ANI of 99.9%. *M. avium* subsp. *avium* had an ANI of 98.7%. ANI of all isolates sequenced in this study including the international isolates were also compared and results are shown in additional file 5.

### SNP identification and phylogenetic analysis of Australian and international map isolates

**Fig. 2:**
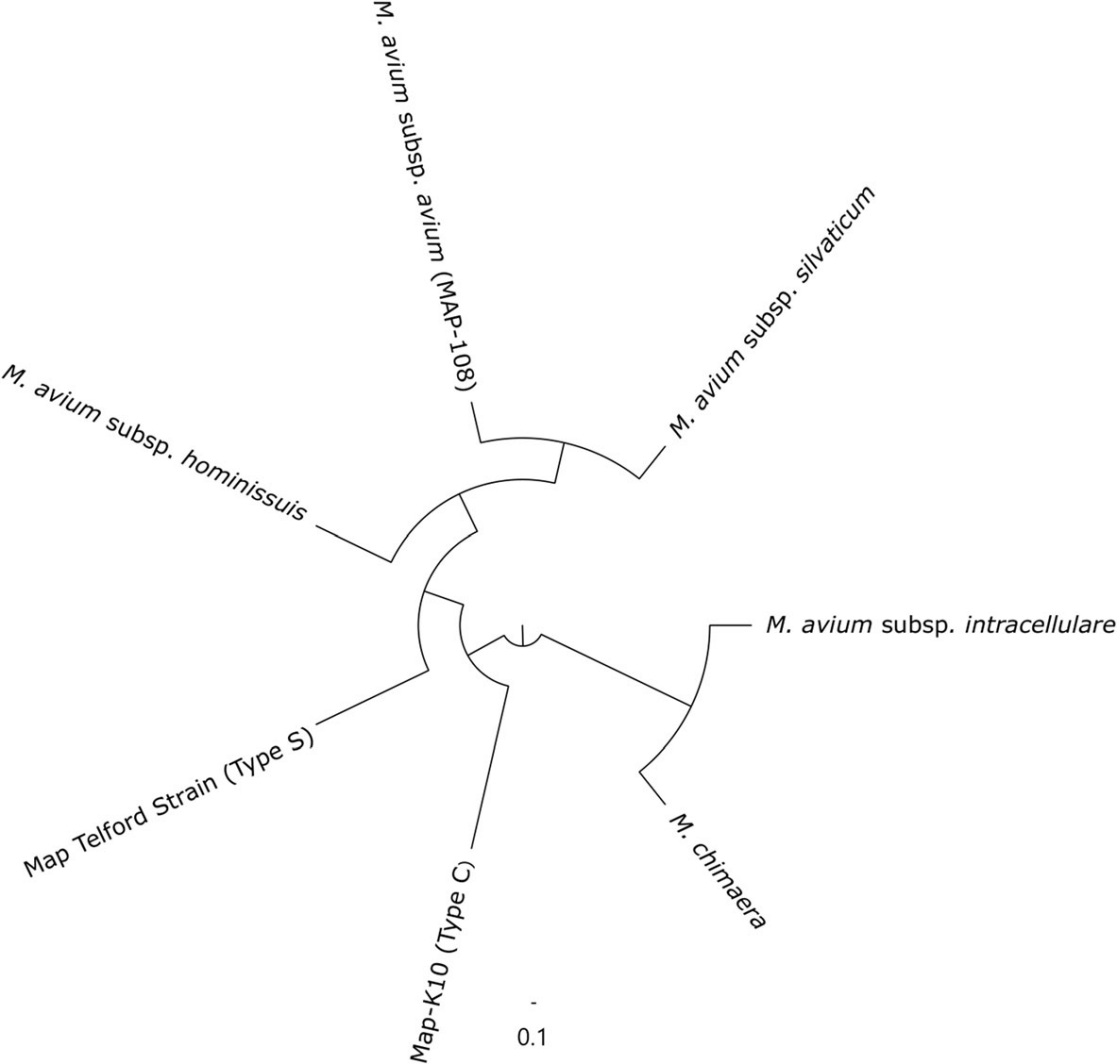
Maximum Likelihood phylogenetic relationship of *Mycobacterium avium* Complex species. The tree is based on core SNPs identified through mapping to Map K10 Strain and rooted at the mid-point

The SNP-based phylogenetic analysis of the core genome of species within the Mac provides an overview of the relationships amongst the members of this cluster of mycobacterial species (Fig. 2). *M. avium* subsp. *avium* is shown to be more closely related to *M. avium.* Subsp. *silvaticum* and *M. avium* subsp. *hominissuis* than to Map.

**Fig. 3:**
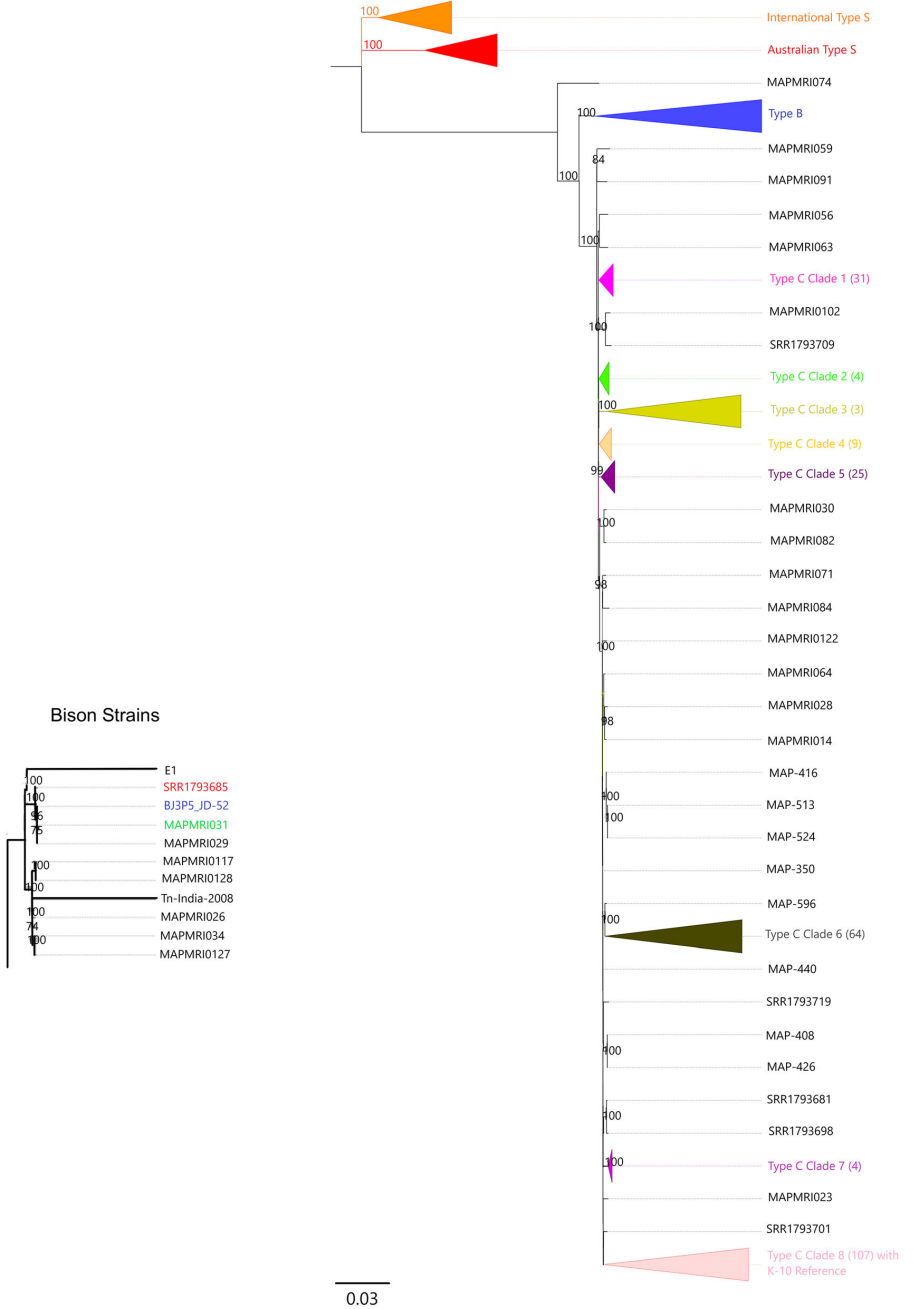
Maximum Likelihood phylogenetic relationship of Australian isolates sequenced in this study including international sequences sourced from NCBI. The tree is based on core SNPs identified through mapping to Map K10 strain and rooted at the mid-point. Branches show bootstrap support and have been collapsed into clades for ease of presentation. The Type B Clade has been expanded and inset to left of the tree. The Australian Type B isolate is highlighted in blue, the Canadian Type B isolate is highlighted in red and the US Type B isolate is highlighted in green

The phylogenetic analysis of SNPs of the core genome identified amongst the Australian and international Map isolates clustered the Type C, the Type S and the Type B strains into distinct lineages all with 100% bootstrap support (Fig. 3). There was an average of 1483 SNPs between Type C strains and Type S strains. The Type S strains all clustered together but formed two distinct clades, one containing all the Australian isolates and one Scottish isolate (MRI0103), while the second clade contained the remaining seven international isolates.

The Type C lineage contained 275 isolates. For ease of viewing, more distinct clusters were collapsed into eight clades (Fig. 3). For all 275 Type C strains, a total of 27,511 SNPs were identified post filtering. There is limited genetic diversity amongst Type C strains with 13–41 SNP differences amongst isolates of each collapsed clade and an average of only 20 SNP differences supporting separation of the eight clades. Each clade had > 99% bootstrap support. There was a total of 28 Australian and international isolates that did not collapse into a clade, each branch still having > 98% bootstrap support. Clades 1, 2, 6, 7, and 8 contained Australian and international isolates, however clades 2 and 7 only had one Australian isolate in each of the clades. Clades 3, 4, and 5 had only international isolates. The only Australian phylogeographical clustering observed in the Type C clades was in Clade 6 where isolates from New South Wales clustered together. Clade 6 also had isolates from the other states across Australia. The AJDRC collection contained six French isolates that did not cluster with other published French isolates that were included in the analysis. Two human isolates (MAP-127 and MAP-160) in the AJDRC clustered together in clade 8 of the Type C strains.

Within the Type B or bison lineage there was 100% bootstrap support between all branches of the tree. The isolates formed three groups which included the US bison strains, the Indian bison strain and one isolate (E1) from Egypt (Fig. 3). There was an average total of 262 SNPs between Type B strains and Type C strains with a total of 14 pairwise SNP differences between bison strains when the Indian bison strain was excluded. The Australian Type B isolate grouped with the USA and Canadian isolates and there was a total of five pairwise SNP difference between the Australian and USA isolate MAPMRI031. The Indian bison strain had a long branch length and a pairwise SNP difference of 578 to Type B strains and 866 SNP differences to Type C strains. Isolate E1 had a long branch length with a pairwise SNP difference of 968 to Type C strains and 286 SNP differences to Type B strains. MAPMRI074, an isolate from a cow in the Netherlands, formed a separate branch to Type C and Type B strains and was more closely related to Type B with a pairwise SNP difference of 440 to Type C and 152 to Type B strains. This isolate was originally identified as Type S by IS*1311* PCR and REA [18] but has been identified as Type C using IS*1311* and LSP analysis in this study.

**Fig. 4:**
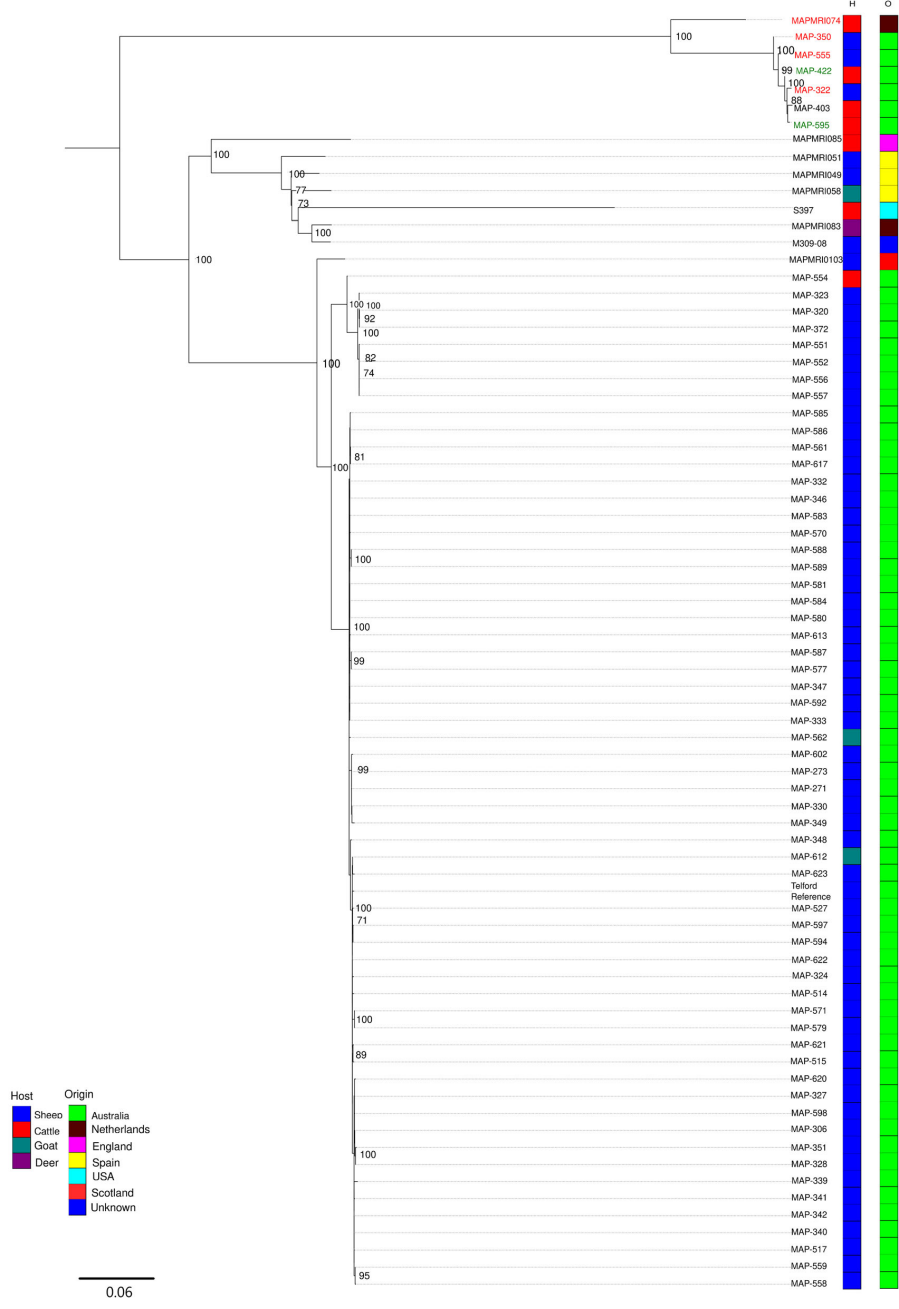
Maximum Likelihood phylogenetic relationship of the 59 Australian Type S isolates sequenced in this study including nine international Type S sequences sourced from NCBI. The tree is based on core SNP’s identified through mapping to MAP Telford strain and rooted at the mid-point. Branches show boot strap support. Host (H) and geographical origin (O) of each isolate is displayed in colour as depicted in the legend. Isolates with names highlighted in red typed as Type S using IS*1311* PCR and REA but have since been identified as Type C and two isolates with names highlighted in green are Type C strains included for comparative purposes

A SNP-based phylogenetic analysis of all 68 Type S isolates, as defined by IS*1311* PCR and REA typing was conducted using the Australian Map-Telford strain [32] as a reference (Fig. 4). This analysis identified a total of 12,089 SNPs amongst Type S strains. All Type S strains formed into two distinct clades: the first clade contained all Australian isolates, as well as one Scottish strain, MAPMRIO103 with 100% bootstrap support; the second clade contained all remaining international isolates with 100% bootstrap support. The average number of SNPs amongst the Australian sheep isolates was 29, whereas there were 764 SNPs among the international isolates. MAPMRIO103 had 177 SNPs different to the Australian Telford reference strain, whereas the other international isolates had an average of 742 SNPs different to the Telford strain (additional file 4, Table S3).

### Identification of IS*1311* and LSP in Australian and international map isolates

To confirm the polymorphism at position 223 in the IS*1311* insertion sequence in all isolates used in this study, sequence data of each isolate was evaluated. Based on this analysis 269 isolates typed as Type C, 62 typed as Type S and eight typed as Type B. In silico analysis of the presence/absence of LSP 4-II, LSP 18, and LSP 20 in all 354 isolates analysed in this study identified 283 isolates as Type C (containing LSP 20), 66 as Type S (containing LSP 18 and LSP 4-II) and five isolates contained all three LSPs therefore a strain type could not be designated.

### In silico SNP based assay for genotyping map

Using the SNP typing system of Leao et al. [13] snp368626, which determines cleavage by the restriction enzyme BsmBI and enables differentiation of Type C and Type S strains of Map, fourteen Type S strains were misidentified as Type C (Table 1). Leao et al. [13] identified Snp4160794, which determines cleavage by the restriction enzyme ApoI, could differentiate between Type C and Type B strains. This typing method was in agreement with the other typing methods for all bison strains analysed in this study. Identification of Snp99782 [13], which is cleaved by restriction enzyme Fat1 for grouping Type C strains into subgroup A and subgroup B, typed all Australian Type C isolates as subgroup A. All the Type C international isolates grouped into subgroup A, except for four isolates: MAPMRI0103, MAPMRI091, MAPMRI059 and MAPMRI026 which were classified as subgroup B.

**Table 1:** Map isolates that generated inconsistent typing results. The Map K10 and Map Telford reference strains are included for comparison

Isolate	Host	Mycobactin Dependency	IS***900***^**a**^ PCR	IS***1311*** PCR Result	IS***1311*** Sequencing Result	LSP-20^**b**^	LSP-18^**b**^	LSP-4^**b**^	LSP Strain Type Result	Phylogenetic Cluster	No. of SNP’s compared to K10 reference	ANI percentage identity compared to K10 reference	SNP based assay of Leao et al [13]
Map K10^c^	Bovine	NT^e^	+	Type C	Type C	+	–	–	Type C	Type C	0	0.999	Type C
Map Telford^d^	Ovine	NT^f^	+	Type S	Type S	–	+	+	Type S	Type S	1533	0.998	Type S
MAP-150	Bovine	+	+	Type B	Type C	+	–	–	Type C	Type C	33	0.999	Type C
MAP-320	Ovine	+	+	Type S	Type S	–	+	+	Type S	Type S	1506	0.998	Type C
MAP-322	Ovine	+	+	Type S	Type C	+	–	–	Type C	Type C	40	0.999	Type C
MAP-323	Ovine	+	+	Type S	Type S	–	+	+	Type S	Type S	1508	0.998	Type C
MAP-328	Ovine	+	+	Type S	Type S	–	+	+	Type S	Type S	1512	0.998	Type C
MAP-339	Ovine	+	+	Type C	Type S	–	+	+	Type S	Type S	1506	0.998	Type S
MAP-340	Bovine	+	+	Type C	Type S	–	+	+	Type S	Type S	1511	0.998	Type S
MAP-350	Ovine	+	+	Type S	Type C	+	–	–	Type C	Type C	33	0.999	Type C
MAP-372	Ovine	+	+	Type S	Type S	–	+	+	Type S	Type S	1505	0.998	Type C
MAP-403	Bovine	+	+	Type S	Type C	+	–	–	Type C	Type C	42	0.999	Type C
MAP-551	Ovine	+	+	Type S	Type S	–	+	+	Type S	Type S	1507	0.998	Type C
MAP-552	Ovine	+	+	Type S	Type S	–	+	+	Type S	Type S	1501	0.998	Type C
MAP-554	Bovine	+	+	Type C	Type S	–	+	+	Type S	Type S	1491	0.998	Type S
MAP-555	Ovine	+	+	Type S	Type C	+	–	–	Type C	Type C	60	0.999	Type C
MAP-556	Ovine	+	+	Type S	Type S	–	+	+	Type S	Type S	1510	0.998	Type C
MAP-557	Ovine	+	+	Type S	Type S	–	+	+	Type S	Type S	1511	0.998	Type C
MAP-571	Ovine	+	+	Type S	Type S	–	+	+	Type S	Type S	1514	0.998	Type C
MAP-588	Ovine	+	+	Type S	Type S	–	+	+	Type S	Type S	1503	0.998	Type C
MAP-598	Ovine	+	+	Type S	Type S	–	+	+	Type S	Type S	1512	0.998	Type C
MAP-612	Caprine	+	+	Type C	Type S	–	+	+	Type S	Type S	1513	0.998	Type S
MAPMRI085	Bovine	NT	+	Type C	Type S	+	+	+	–	Type S	1463	0.998	Type S
SRR1793685	Bovine	NT	+	Type C	Type B	+	–	–	Type C	Type B	290	0.999	Type C
MAPMRI049	Ovine	NT	+	Type S	Type S	–	+	+	Type S	Type S	1442	0.998	Type C
MAPMRI058	Caprine	NT	+	Type S	Type S	–	+	+	Type S	Type S	1474	0.998	Type C
MAPMRI074	Bovine	NT	+	Type S	Type C	+	–	–	Type C	Type C	440	0.999	Type C
MAPMRI083	Cervine	NT	+	Type S	Type S	–	+	+	Type S	Type S	739	0.998	Type C
MAPMRI051	Ovine	NT	+	Type S	Type S	–	+	+	Type S	Type S	1464	0.998	Type C
MAPMRI094	Ovine	NT	+	Type S	Type S	–	+	+	Type S	Type S	88	0.998	Type C

^a^IS*900* PCR is reported to be specific for identifying Map

^b^ LSP-20 is present in type C strain and absent in type S, LSP-18 is present in type S strain and absent in type C strain, LSP-4 is present in type S strain and absent in type C

^c^Map K10 reference strain (NC_002944.2), IS*900* PCR and IS*1311* PCR and REA results were obtained from published data [33, 34]

^d^Map Telford reference strain (NZ_CPO33688.1), IS*900* PCR and IS*1311* PCR and REA results obtained from published data [35]. Type strains included for comparative purposes

^e^+ = positive result, − = negative result, blank space = result unknown

^f^NT = Not tested in this study

### Comparison of identification and strain typing methods

All Australian Map isolates sequenced in this study were confirmed to be Map by IS*900* PCR [36]. However, the IS*900* insertion sequence was found not to be present in the assembled genome of Map-116 further indicating that it is likely that the original isolate was co-cultured with Map and that MAP-116 was better preserved in storage and the dominant isolate recovered for further analysis. BLASTn [37] analysis indicated that *M. chimaera* was the closest relative to MAP-116 with the majority of assembled contigs having a > 90% sequence identity (data not shown). Three other isolates, MAP-107, MAP-115 and MAP-119 were also IS*900* PCR positive but strain typed as *M. avium* subsp. *avium* by IS*1311* PCR. The IS*900* insertion sequence was also found not to be present in the assembled genomes of these isolates, again indicating that the isolates were likely to be a co-culture of Map and *M. avium* subsp. *avium*. A comparison of all the strain typing methods is presented in Table 1. There were 32 isolates (24 Australian and eight International) that did not have full agreement amongst typing methods. One international isolate, MAPMRIO85 contained LSP-20, LSP-18 and LSP-4 and therefore no strain type could be inferred from these results using the LSP typing method. The IS*1311* PCR and REA typing methods typed MAPMRIO85 as Type C strain, while phylogenetic analysis clustered MAPMRIO85 with Type S strains. MAP-150 typed as a Bison strain based on IS*1311* PCR and REA analysis but identified as a Type C strain based on presence/absence of LSP genes. This isolate clustered with Type C strains in phylogenetic analysis and had an ANI of 0.999 compared with K-10 Type C reference strain versus an ANI of 0.998 compared to Type S strains. Another Bison strain, SRR1793685, typed as a Bison strain based on IS*1311* sequencing and phylogenetic analysis but is classified as a Type C strain based on IS*1311* PCR and REA and presence/absence of LSP genes. Four other isolates, MAP-339, MAP-340, MAP-554, and MAP-612, classified as Type C strains based on IS*1311* PCR and REA analysis and as Type S strains based on LSP and phylogenetic analysis and ANI. Isolates MAP-322, MAP-350, MAP-403, MAP-555, and MAPMRIO74 were classified as Type S using IS*1311* PCR and REA analysis, and Type C based on the presence/absence of LSPs and clustered with Type C strains in phylogenetic analysis. Map-320, MAP-323, MAP-328, MAP-372, MAP-551, MAP-552, MAP-556, MAP-557, MAP-571, MAP-588, MAP-598, MAPMRI051, MAPMRI094, MAPRI049, MAPRI048 and MAPRI083 all typed as Type S by all methods except for the SNP based assay method by Leao et al. [13] which typed these isolates as Type C.

## Discussion

This study represents the first phylogenetic and thorough comparative typing analysis for assessment of the genetic diversity of Australian Type C, Type S and Type B Map isolates in the context of international strains. This is the largest comparative genomic study of Type S Map strains to date with most genetic diversity studies of Map predominately focusing on cattle strains [18, 38]. The phylogenetic analysis of *Mycobacterium avium subsp. avium, M. avium* subsp*. hominissuis* and *Mycobacterium avium subsp. silvaticum* in this study showed that they were phylogenetically more closely related to each other than Map as has been previously suggested in other studies [28, 39, 40]. An Australian isolate MAP-116 was originally identified as Map because it was IS*900* PCR positive and mycobactin dependent, however further analysis revealed as the IS*900* region was not present in the genome sequence and that this isolate was most likely co-cultured with a low titre Map and the dominant organism that survived better in storage. Analysis of the IS*900* sequence in the genomic data further revealed that the IS*900* insertion sequence was not present in MAPMRI094. MAPMRIO94 is a Type S strain of Map and a previous study has found that some copies of IS*900* have undergone polymorphisms in some sheep strains [41]. In the past, the presence of the IS*900* insertion sequence was considered to be unique to Map and targeted by the IS*900* PCR to differentiate Map from other species of *Mycobacterium* [42]. However, Cousins et al. [43] identified environmental mycobacterium isolates that were IS*900* PCR positive, but these could be differentiated from Map on the basis of mycobactin dependence, a feature that can be used as a confirmatory test for the positive identification of Map in culture [44]. Species of Mycobacteria share very similar ecological niches such as water, soil, wastewater, protozoa, deep litter, animals and humans [45] which increases the risk of co-infection within an animal host. Evidence of co-infection of Map with other species of Mycobacteria in cattle already exists and has been shown to interfere with diagnosis. For example, co-infection of Map with *M. bovis* has been shown to have an effect on the immunological response to tuberculins leading to false negative results [46] and affecting the sensitivity of the *M. bovis* skin test and interferon gamma assay [47]. This could be problematic for the diagnostic laboratory especially if one mycobacterium outgrows the other and the incorrect colony type is selected causing false negative results. In some cases, an IS*900* PCR positive result may still be obtained, however with or without co-infection relying solely on an IS*900* PCR result without showing mycobactin dependency may lead to false positive results.

BLASTn analysis indicated that MAP-116 was most closely related to *M. chimaera*. *M. chimaera* is a slow growing mycobacterium found in soil and water [48]. It has recently been found to cause infection in patients undergoing heart surgery due to contamination of heating-cooling units [49]. As MAP-116 was likely to be co-cultured with Map it is the intention of the authors to do further work on isolating the bacteria and more in-depth analysis of the genome and phenotype to fully characterise the isolate as *M.chimaera* has not been isolated from an animal source in the past.

Phylogenetic analysis of Type C strains revealed how genetically monomorphic Map is globally. There was limited phylogeographical clustering of Map within Australia or internationally as the international strains were dispersed throughout the Australian strains. These findings suggest multiple introductions of Map into Australia and that there is significant movement of infected dairy cattle throughout Australia and the world.

The only phylogeographic trend observed amongst Australian isolates in this analysis was that all NSW isolates clustered together within Clade 6 of the Type C isolates, together with other Australian isolates and one Canadian isolate. All nine of the NSW isolates were isolated in 2016 and are likely to represent a single outbreak. There was a total of 13 pairwise SNP differences within clade 6 which would indicate that the isolates share a recent common ancestor.

Within the Type C clades, clade 3, 4 and 5 contained only international isolates and although they were from various countries throughout Europe and Canada, there were subgroups where isolates from the same country clustered together, suggesting some phylogeographic relationship. A similar relationship of clustering within subgroups occurred in clade 8 of the Type C strains which contained both Australian and international isolates. Within the AJDRC there were six Type C isolates that originated from France. Four of those isolates clustered together in clade 1, the same clade as other French isolates from the international collection analysed in this study, however they did cluster into a subgroup separate to the other French isolates. This may suggest that they share the same ancestor but originated from a different herd in France.

Two human isolates in the AJDRC that were isolated nine years apart clustered together in clade 8 within the Type C lineage. Unfortunately, there is no clinical information about the patients from which they were isolated (e.g. whether these isolates originated in Crohn’s disease patients). However, these isolates typed as Type C which is the same strain type as other Map isolates that have been isolated from Crohn’s disease patients [7, 50, 51].

All Type B strains clustered together and were closely related to Type C strains (Fig. 3). In this study the bison isolates formed into three phylogenetic groups: the US bison type, the Indian bison type and one isolate from Egypt (E1). The Australian Type B isolate, BJ3P5_JD-52, was most closely related to the US Type B strain which would suggest that they have derived from a common ancestor. This isolate was isolated from a cow in Victoria, whereas the international isolates were from bison herds. The prevalence of Type B Map in Australia needs to be determined to better understand the evolutionary history of Type B strains in Australia. The Indian-Bison type had a long branch length and a pairwise SNP difference between 569 and 583 to the other Type B isolates indicating that the Indian-Bison type is more genetically diverse. The isolate E1 from Egypt had previously been typed by IS*1311* PCR and REA as a Type C strain [52], however the authors also found that the E1 strain had a higher number of SNPs to the K-10 reference strain than other Type C isolates. In this study E1 clustered more closely with Type B strains and not Type C strains with a pairwise SNP difference of between 671 and 685 to Type B strains in comparison to a pairwise SNP difference of 968 to the Map K10 Type C strain. In silico analysis of the E1 strain found that it typed as Type B using the SNP based assay developed by Leao et al. [13]. Type B strains have not been well studied and therefore the evolutionary history of the bison strain is not fully understood. Type B strains were first isolated from Bison in Montana in the USA and were originally thought to be Type C strains until their cultural characteristics and disease manifestation in animals was seen to be different [17, 18]. Further studies of the IS*1311* sequence showed differences between Type C and Type B strains and that the IS*1311* sequence was consistent across all Type B strains. A third group of Type B isolates, the Indian-Bison type was identified with differences within the IS*1311* sequence that were also consistent within that group [53].

There was a total of 68 Type S strains sequenced in the study, of which 59 came from the Australian Reference collection and nine were international isolates. Phylogenetic analysis of Type S strains identified two distinct clades; a clade that included all Australian isolates and one Scottish isolate and a clade of all other international isolates, suggesting a single introduction of the Type S strain into Australia. Johne’s disease in sheep was initially diagnosed in Australia in New South Wales in 1980 [54]. The origin of this outbreak was never determined but was thought to be linked to the importation of carpet-wool sheep from New Zealand between 1975 and 1979 [55]. However, there is empirical evidence from government veterinarians from the 1960’s and 70’s to suggest that it is more likely that Type S strains have been present in Australia since the 1950’s [56]. A national disease control program for Ovine Johne’s disease (OJD) began in Victoria in 1997 which involved destocking of infected properties [57] and resulted in Western Australia being declared OJD free in 1999 [58]. A National Bovine Johne’s Disease strategic plan was not implemented until 2003 [57] which may explain why it is more likely that there were multiple introductions of Type C Map but not Type S Map into Australia. Due to the difficulties associated with culturing Map Type S prior to the introduction of 7H10 liquid medium [59], there are limited early Australian Map Type S isolates available for analysis to confirm the epidemiology of Type S in Australia.

Although this is the first study of Type S strains of Map from within Australia, there were no NSW isolates for comparison which in addition to Western Australia and Victoria, contributes significantly to the sheep industry in Australia. There have also been limited diversity studies of Type S strains internationally. More genetic data from NSW and international isolates is required to determine the origin of the Australian Type S strains of Map. Within the Australian Type S isolates there were isolates that originated from Victoria, Queensland and Western Australia and these isolates were dispersed throughout the Type S clade. Isolates that were from the same geographical region that had similar collection dates did cluster together within the clade, indicating they were most likely from the same outbreak. The dispersion of Map isolates throughout Victoria, New South Wales, Queensland and Western Australia is consistent with the well-known frequent and extensive movement of sheep across the country [60].

Type C strains do not have a host species preference and can be found in a broad range of ruminants and non-ruminants [61]. Conversely Type S strains are thought to have some host-preference being predominately isolated from sheep [15]. However, clinical disease has been induced in cattle experimentally infected with Type S [62]. There have also been some reports of natural infection of Type S strains in cattle, wild deer and Arabian camelids [63]. According to results obtained in this study there has been little cross species transmission of Map strains in domesticated Australian ruminants. Australian Type C strains were predominately isolated from cattle with only four isolates out of 165 being isolated from other animal hosts, including two isolates from humans. Australian Type S strains from the AJDRC were also predominately isolated from sheep with only one out of 59 isolated from a cow. The international Map isolates that were included in this study indicate that cross species transmission may be more common globally than in Australia, however this may reflect the type of strains that have been sequenced rather than a true indication of the prevalence of cross species transmission. However, there is a larger population of both sheep and cattle in some of these countries that are commonly grazed together which may give rise to greater opportunity for cross species transmission. There has also been compelling evidence of cross species transmission in New Zealand [18] and dating back to the 1930s in Iceland [64]. In Scotland it has been reported that rabbits and carnivores have been infected with Map [65]. There have been no studies of cross species transmission conducted in Australia although rabbits, kangaroos and other wildlife are seen co-grazing with sheep and cattle, and they could act as reservoirs of Map and be a source of cross species transmission..

ANI results in this study supported the lack of genetic diversity of Map as Type C and Type B strains had an identical ANI value and there is only a 0.02% ANI difference when Type C strains are compared to Type S strains. The advantages of using ANI to determine species relatedness within a genus is that it is simple, easy to interpret, is more robust and has a higher power of resolution for phylogenetic relatedness [66]. ANI for most bacterial genera has an accepted value of 96% with a coverage of > 90% over the genome for accurate identification to the species level. Acceptance criteria can be adjusted to accommodate unusually monomorphic species (eg. Map, *B.anthracis*) to reflect a clearer relationship between species or subspecies within a genus [67]. Within the *Mycobacterium* genus it has been proposed that a true match to the same species should have an ANI > 99.8% with 98% coverage [67]. All sequences used in this study had a coverage of > 98% to Map K10. ANI relatedness values were > 99.8% for Type C, Type B and Type S strains. *M. avium* subsp. *avium* had an ANI of 98.7% indicating a close relatedness to Map. ANI values correlated with phylogenetic analysis for all Map isolates as shown in Fig. 1.

Monomorphic bacteria such as Map can be very difficult to differentiate between strain types due to the very low levels of polymorphisms found in their genomes [14]. IS*1311* genotyping is the most commonly used method for strain typing of Map in many laboratories. The IS*1311* insertion sequence is present in both Map and *M. avium* and specifically the point mutation at nucleotide position 223 of IS*1311* has been found to be present in Type C strains of Map but absent from Type S strains [19]. In this study, IS*1311* genotyping was compared to ANI, phylogenetic inference-based analysis using core SNPs, LSP presence/absence and in silico analysis of IS*1311* insertion sequence and showed that the IS*1311* genotyping results were not always in agreement with the other typing methods evaluated.

The phylogenetic analysis based on core SNPs identified through comparison to the K-10 and Telford reference strains supported typing via LSP presence/absence, and in silico IS*1311* sequencing results. In a previous study IS*1311* genotyping incorrectly identified a Type C strain as a Type S strain suggesting that the allelic variation to differentiate the strains occurred after the Type C strain initially diverged from the Type S strain [18]. However, in this study IS*1311* genotyping incorrectly typed isolates of all three strains (C, S and B) and typed an isolate that was not Map, suggesting that the IS*1311* insertion element is an unsuitable genetic marker for strain typing Map isolates. It is widely reported that sequence variants that are detected within IS elements are one of the most rapidly evolving within the microbial genome [14], and therefore may not be the most suitable method to use for strain differentiation. Strain differentiation using IS*1311* genotyping has proven to be a difficult method to distinguish between Type B and Type C strains of Map. This method relies on the presence of a T at position 223 in all copies of the IS*1311* insertion sequence for Type B and a C or T at position 223 in only one or more copies for Type C. One isolate in this study was incorrectly identified as a Type B using this method. In silico analysis of the IS*1311* insertion sequence found that there was an insertion or deletion at position 63 in all Type B isolates that was not present in Type C or Type S strains. Therefore, a SNP based assay targeting a stable region of the genome rather than the currently used IS*1311* PCR and REA may be a more reliable method to differentiate between Type C and Type B strains.

SNP typing has increasingly been used for differentiation of strains within bacterial species as well as phylogenetic analysis of monomorphic bacteria [13, 14]. A SNP based assay for differentiating Map using 14 SNPs and which requires 14 PCR and REA reactions to be performed, was recently developed to differentiate between Type C, Type S and Type B strains and subgroup Type C strains [13]. In silico results from the 356 isolates analysed in this study found these 14 SNPs to be unreliable for differentiation of Type S strains. The primer pair used by Leao et el [13] to differentiate between Type C and Type S misidentified 21 isolates that were identified as Type C rather than Type S. All Australian Type C isolates were sub-grouped into subgroup A using this SNP assay and all bison strains typed as Type B. More isolates from all strain types on a global scale need to be analysed to better characterise isolates in order to have a better SNP based assay that will differentiate Map strains.

## Conclusions

This is the first study that has investigated the phylogeny of Map isolates in Australia using whole genome sequencing and SNP analysis and their relationship compared to an international collection of Map isolates. It is also the first in-depth comparison of typing methods of Map. It represents the largest study of Type S strains investigated to date. The results show that Map is extremely monomorphic with very little differences in ANI between strain types. Within Type C strains, Australian and international isolates were dispersed throughout the clades indicating that there is limited phylogeographic clustering of Type C Map within Australia or at an international level. Australian isolates from different states were interspersed throughout the whole genome SNP based tree suggesting that there is significant movement of cattle and therefore Map throughout the states. Type S strains separated into two distinct clades with Australian isolates and one Scottish isolate in one clade and international isolates in the other. In contrast to cattle this suggests that there is some phylogeographical clustering of Type S strains. Australian Type S strains were most closely related to an isolate from Scotland, however more Australian and international Type S strains need to be sequenced and compared to shed light on the origin of Australian Type S strains. The importation of sheep into Australia ceased a few decades ago due to transmissible spongiform encephalopathy (TSE) and now only embryos and semen can be imported into the country. This ban on sheep importation may have influenced the phylogeographical clustering observed in sheep. In comparison, cattle have not been imported into Australia for about 20 years from North America, Japan and Europe which may explain why cattle isolates were interspersed amongst international strains. The Australian Type B strain was closely related to a US bison herd, but more Australian isolates of the Type B strain need to be sequenced to fully determine the origin of Type B strains in Australia. Four Australian isolates, MAP-116, MAP-107, MAP-115 and MAP-119 were all IS900 PCR positive, however the IS900 insertion sequence was not present in their assembled genomes indicating there was likely to be co-inoculation of Map with these isolates. MAP-107, MAP-115 and MAP-119 typed as *M. avium* subsp. *avium* and phylogenetically were more closely related to *M. avium* subsp. *avium,* whereas MAP-116 was more closely related to *M.chimaera* based on blastn analysis. The comparison of different strain typing methods indicated that IS*1311* genotyping is an unreliable method for strain differentiation of Map, while LSP presence/absence, phylogenetic analysis, ANI and SNP analysis were all in agreement for determining strain type. This comprehensive study of Australian Type C, Type S and Type B strains has resulted in genomic information that will allow the development of alternative methods for strain differentiation of Map. It is our intention to use this information to develop a SNP-based assay in the future.

## Methods

### Panel of strains

Australian Map isolates used in this study were obtained from the Australian Johne’s Disease Reference Collection (AJDRC) held at The Department of Jobs, Precincts and Regions, AgriBio Centre, Victoria, Australia. These isolates collected over a 35-year period, represent individual faecal or tissue samples from different locations in Victoria, New South Wales, Queensland, Tasmania, Western Australia and six isolates from France. There was a total of 228 isolates from the collection, of which 157 were derived from cattle, 58 from sheep, three camelid, six caprine, one cervine, one avian and two human isolates. All isolates within the AJDRC collection were recovered and included in this study except where there were multiple isolates from the same animal or farm. Four *Mycobacterium avium subsp. avium* isolates were also included in the study as an outgroup. The geographical location, host and year of isolation of the Australian and international isolates are presented in additional file 1, Table S1. A total of 172 Map isolates from the AJDRC (two Type B, 166 Type C and four *M. avium*) were propagated on Middlebrook 7H10 agar supplemented with Middlebrook oleic acid-albumin-dextrose-catalase (OADC) and 2 mg ml^− 1^ mycobactin J [68], as well as Middlebrook 7H10 agar supplemented with Middlebrook oleic acid-albumin-dextrose-catalase (OADC) and no addition of mycobactin to confirm mycobactin dependency [68]. All 59 sheep strain isolates were propagated in Middlebrook M7H9C broth supplemented with Middlebrook oleic acid-albumin-dextrose-catalase (OADC) and 2 mg ml^− 1^ mycobactin J [59]. The isolates were incubated at 37 °C until colonies could be visualised on slopes and 12 weeks in broth. Single colonies from the Middlebrook 7H10 agar were sub-cultured for subsequent use. In addition, genotyping information and sequence data from 123 strains of Map from diverse geographic origins were downloaded from the European Nucleotide Archive Database and the SRA database from the National Center for Biotechnology Information (NCBI). All information pertaining to these isolates are as previously described [18, 38]. Sequence data from four subspecies within the Mac (*M. avium chimaera* AH16, accession PRJNA294790; *Mycobacterium intracellulare*, accession CPO23149; *Mycobacterium avium subsp. hominissuis*, accession CPO40247; and *Mycobacterium avium subsp. silvaticum*, accession PRJEB2204) were also downloaded from NCBI.

### Preparation of DNA

Genomic DNA was extracted using the Wizard Genomic DNA Purification Kit (Promega, Madison, WI, USA) with some modifications. Map isolates grown on Middlebrook 7H10 agar were harvested in 1 ml of sterile distilled water and centrifuged at 13,000 x g for 2 min. Pellets were resuspended in buffer containing 200 μl Ethylenediaminetetraacetic acid (EDTA), 120 μl 1.2% Triton-X and 200 mg of Lysozyme and incubated in a 37 °C water bath for 1 h, centrifuged at 16,000 x g for 2 min and supernatant removed. The pellet was resuspended in 600 μl of Nuclei lysis solution and 20 μl of Proteinase K and incubated at 55 °C for 10 min, followed by an 80 °C incubation for 5 min, and the manufacturers protocol was followed from step 8 (Promega, Madison, WI, USA). For isolates grown in M7H9C broth, 1 ml of culture was centrifuged at 13,000 x g for two minutes, pellets were then washed twice by resuspending pellet in 1 ml of sterile distilled water and centrifuging at 13,000 x g for 2 min and DNA extracted as described above.

### IS*900* PCR

IS*900* PCR [36] was used for identification of all Map isolates from the AJDRC and used in this study (additional File 1: Table S1). The PCRs were performed using 1 μl of extracted DNA in a PCR mix containing 1 μM of primers IS*900*/150C and IS*900*/921, 10 mM Tris hydrochloride, (pH 8.3), 50 mM potassium chloride, 1.5 mM magnesium chloride, 0.01% gelatin, 200 μM of each of the nucleotides dATP, dTTP, dGTP and dCTP, and 2.5 U of Taq polymerase. The assay was performed on a Veriti 96 well thermo cycler (Thermo Fischer Scientific, Massachusetts, USA) under the following conditions: 35 cycles of denaturation at 94 °C for 10 s, annealing at 60 °C for 10 s, and extension at 72 °C for 10 s. The expected 229 bp IS*900* PCR amplicon was visualised on a 2% agarose gel stained with Syber Safe (Invitrogen).

### IS*1311* PCR and REA analysis

IS*1311* PCR and restriction endonuclease analysis (REA) was used for strain characterisation of all Map isolates used in this study from the AJDRC (additional File 1: Table S1). For the IS*1311* PCR 5 μl of extracted DNA was used in a PCR mix containing 250 ng of each primer M-56 and M-119, 200 μM of each of the nucleotides dATP, dTTP, dGTP and dCTP, 10 x PCR buffer (Qiagen, Germany), 25 mM magnesium chloride (Qiagen, Germany) and 2 U of HotStar Taq polymerase (Qiagen, Germany) [69]. The assay was performed on a Veriti 96 well thermo cycler (Thermo Fischer Scientific, Massachusetts, USA) under the following conditions: initial denaturation of 94 °C for 3 min, and then 37 cycles of denaturation at 94 °C for 30 s, annealing at 62 °C for 15 s, and extension at 72 °C for 1 min. The REA was performed to detect the C/T polymorphism at base pair 223 of the IS*1311* gene by adding 5 μl of IS*1311* PCR product to a total reaction mix of 16 μl containing 2 U of HinfI and 2 U of MseI supplemented with 10 x NE buffer (New England Biolabs, Massachusetts, USA) and 10 x BSA (Promega, Madison, WI, USA). The restriction digest was incubated for 3 h at 37 °C. After 3 h the DNA fragment patterns were visualised as bands on a 3% agarose gel stained with Syber Safe. Isolates were designated as Type C if 67 bp, 218 bp, 285 bp and 323 bp fragments were generated, Type S if 285 bp and 323 bp fragments were generated, Type B if 67 bp, 218 bp and 323 bp fragments were generated and *M. avium* if 134 bp, 189 bp and 285 bp fragments were generated.

### Genome sequencing, assembly and average nucleotide identity

Genomic DNA libraries were prepared for all Australian isolates using an Illumina Nextera™ DNA Flex Library Prep kit with CD indexes according to the manufacturer’s instructions (Illumina, San Diego, CA, USA). The size distribution of libraries was determined using the HSD1000 ScreenTape device on the 2200 TapeStation system (Agilent technologies). Concentration was determined using the Qubit Fluorometer2.0 (Invitrogen) and KAPPA Library Quantification kit (KapaBiosystem) according to the manufacturer’s instructions. Pooled libraries were sequenced (2 × 250 bp paired end reads) using a Miseq v3 reagent kit on an Illumina Miseq® platform (Illumina, San Diego, CA, USA) according to the manufacturer’s instructions. Illumina reads were quality trimmed using Trimmomatic [70] with the following parameters: > 15 quality score, sliding window of 4 and minimum length of 200 bp.

ANI between all possible pairs of 50 representative assembled genomes was calculated using pyani [71] and visualised using Pheatmap v1.2.12 in R. Quast version 5 was used to evaluate the assemblies and provide genome metrics including contig number, GC content and N50 values [72].

### SNP identification and phylogenetic analysis

Quality checked reads from 351 isolates of Map were aligned to Map K10 type strain (NC_002944.2) as a reference genome using bowtie2 v2.3.5.1 [73] as part of the RedDog pipeline version VIbeta.10.3 [74] with default parameters. Repetitive regions were identified in the reference Map K10 genome using Red [75] and then removed to minimise false positives. Additional variant calling was performed on the bam files using freebayes v1.3.1 [76] with filtering criteria that ensured a minimum depth > 10 and a quality score > 200. A maximum likelihood phylogenetic tree was inferred using RAxML version 8.2.11 [77] using a GTRGAMMA model with 1000 bootstrap replicates. The resulting RAxML best tree was visualised in FigTree version 1.4.4 [78]. Clades were selected by capturing the majority of subpopulations sharing a branch. All Type S isolates were also aligned to Map Telford strain (NZ_CPO33688.1) as a reference as described above. A selection of subspecies from Mac were also aligned to the Map K10 (NC_002944.2) as a reference genome as described above and each data set were subjected to the same procedures for variant calling and phylogenetic tree construction.

### *In-silico* identification of IS*1311* and long sequence polymorphism

To confirm which Map sequences had the same polymorphism at position 223 the IS*1311* sequence of all isolates was aligned to *M. avium* U16276 using bowtie2 v2.3.5.1 [73]. Bamtools v2.5.1 and freebayes v1.3.1 [76] were then used to create variant calling format files (vcf) listing the IS*1311* polymorphisms present in each Map isolate. An alignment was performed using MUSCLE v3.8.31 [79] to show position 223 within the IS*1311* sequence of select isolates that depict variation present, including the presence of different SNPs in IS*1311* in the same isolate (additional File 2: Fig. S1).

De novo assembly of quality trimmed reads was performed for each isolate using Unicycler v0.4.7 [80] using default parameters.

The sequence for each LSP: LSP 4-II, LSP 18, and LSP 20, was identified using Primer-BLAST [81] with primers that had previously been designed for amplification of the LSPs in *Mycobacterium avium sp.* [82]. Using blastn [37] these amplicon sequences were aligned to all Map isolates analysed in this study to confirm the presence/absence of these LSP segments, IS900 and sequence identity to species of Mycobacteria.

### In silico analysis of SNP based assay for strain differentiation

A novel SNP based assay which utilises 14 discriminative SNPs obtained through phylogenetic analysis of 133 Map isolates has been developed by Leao et al. [13] to differentiate between Type C and Type S strains, Type C and Type B strains and to subgroup Type C strains. To determine if this assay could be used to differentiate Australian isolates the target regions of the three primer pairs used to differentiate between Type C and Type S strains, Type C and Type B strains and subgroups of Type C strains were identified using Primer-BLAST [81]. The target sequence for each primer set was then uploaded to RestrictionMapper Ver 3.0 and the specific enzyme corresponding to each target was selected to identify the restriction sequence and the location of the restriction site. The vcf files from the reference genome alignment were examined to identify variants in the region of the restriction enzyme target site. An alignment was created using the Map K-10 reference sequence for each target and visualised using Tablet v1.17.08.17 [83].

## Supplementary Information

**Additional file 1 **: **Table S1.** Isolate name, host, location, year, PCR, LSP and IS*1311* and REA results of all isolates that were analysed and used for phylogenetic analysis in this study.

**Additional file 2 **: **Figure S1**. Muscle alignment of the IS*1311* insertion from *M. avium* subsp. *avium* (U16276.1), Map K10 (NC_002944.2), MAP 4 (NC_021200.1), and Telford (CP033688.1) that contains the SNP at position 223 targeted by the restriction enzyme analysis (REA).

**Additional file 3 **: **Table S2.** Australian and International Map isolates that showed cross species transmission.

**Additional file 4: Table S3.** Sequencing depth and coverage, number of SNP’s before and after filtering when aligned to Map K10 and Telford strain, GC content, number of contigs and number of raw sequence reads generated from the next generation sequencing of isolates that were sequenced and used for phylogenetic analysis in this study against the K10 reference strain

**Additional file 5 **: **Table S4.** Pairwise comparison of average nucleotide identity (ANI) values of all *Mycobacterium* isolates included in this study.

## Data Availability

The datasets generated and/or analysed during the current study are available in the NCBI Bioproject repository, accession number PRJNA632696 (https://www.ncbi.nlm.nih.gov/sra/PRJNA632696).
