# Supplementary material for: Molecular characterisation of Mycobacterium avium subsp. paratuberculosis in Australia
Source: BMC Microbiol. 2021 Apr 1;21:101. doi: 10.1186/s12866-021-02140-2 (PMC8012159; doi:10.1186/s12866-021-02140-2)
Supplement: Supplementary file 1 — Additional file 1 : Table S1. Isolate name, host, location, year, PCR, LSP and IS1311 and REA results of all isolates that were analysed and used for phylogenetic analysis in this study. [file 12866_2021_2140_MOESM1_ESM.docx]

**Additional file 1: Table S1.** Isolate name, host, location, year, PCR, LSP and IS1311 and REA results of all isolates that were analysed and used for phylogenetic analysis in this study

| **Isolate** | **Host** | **Location** | **Year** | **IS900** | **Type C Clade** | **LSP-20** | **LSP-18** | **LSP-4** | **IS1311/REA** |
| --- | --- | --- | --- | --- | --- | --- | --- | --- | --- |
| MAP-102 | Bovine | Victoria | 1996 | +ve | 8 | + | - | - | +ve, Cattle |
| MAP-106 | Bovine | Victoria | 1996 | +ve | 8 | + | - | - | +ve, Cattle |
| MAP-107 | Bovine | Victoria | - | +ve | - | + | + | + | +ve, Avium |
| MAP-108 | Avian | Victoria | - | +ve | - | + | + | + | +ve, Avium |
| MAP-112 | Bovine | Victoria | 1991 | +ve | 8 | + | - | - | +ve, Cattle |
| MAP-113 | Bovine | Victoria | - | +ve | 7 | + | - | - | +ve, Cattle |
| MAP-114 | Bovine | Victoria | 1991 | +ve | 8 | + | - | - | +ve, Cattle |
| MAP-115 | Bovine | Victoria | 1996 | +ve | - | + | + | + | +ve, Avium |
| MAP-116 | Camelid | Victoria | 1996 | +ve | - | + | + | + | +ve, Cattle |
| MAP-118 | Bovine | Tasmania | 1985 | +ve | 6 | + | - | - | +ve, Cattle |
| MAP-119 | Bovine | Victoria | - | +ve | - | + | + | + | +ve, Avium |
| MAP-120 | Bovine | Victoria | - | +ve | 6 | + | - | - | +ve, Cattle |
| MAP-121 | Caprine | France | 1989 | +ve | 2 | + | - | - | +ve, Cattle |
| MAP-122 | Bovine | Victoria | 1995 | +ve | 6 | + | - | - | +ve, Cattle |
| MAP-123 | Bovine | Victoria | 1995 | +ve | 8 | + | - | - | +ve, Cattle |
| MAP-125 | Bovine | Victoria | 1996 | +ve | 8 | + | - | - | +ve, Cattle |
| MAP-127 | Human | Victoria | 1984 | +ve | 8 | + | - | - | +ve, Cattle |
| MAP-128 | Bovine | Tasmania | 1987 | +ve | 6 | + | - | - | +ve, Cattle |
| MAP-129 | Ovine | France | 1989 | +ve | 8 | + | - | - | +ve, Cattle |
| MAP-131 | Bovine | Victoria | 1993 | +ve | 6 | + | - | - | +ve, Cattle |
| MAP-132 | Bovine | Victoria | 1993 | +ve | 8 | + | - | - | +ve, Cattle |
| MAP-134 | Bovine | Victoria | 1993 | +ve | 6 | + | - | - | +ve, Cattle |
| MAP-135 | Bovine | Victoria | 1993 | +ve | 6 | + | - | - | +ve, Cattle |
| MAP-138 | Bovine | Victoria | 1996 | +ve | 8 | + | - | - | +ve, Cattle |
| **Isolate** | **Host** | **Location** | **Year** | **IS900** | **Type C Clade** | **LSP-20** | **LSP-18** | **LSP-4** | **IS1311/REA** |
| MAP-139 | Camelid | Victoria | 1996 | +ve | 8 | + | - | - | +ve, Cattle |
| MAP-140 | Bovine | Victoria | 1995 | +ve | 6 | + | - | - | +ve, Cattle |
| MAP-141 | Bovine | Victoria | 1996 | +ve | 6 | + | - | - | +ve, Cattle |
| MAP-142 | Bovine | Victoria | 1996 | +ve | 6 | + | - | - | +ve, Cattle |
| MAP-143 | Bovine | Victoria | 1996 | +ve | 1 | + | - | - | +ve, Cattle |
| MAP-144 | Bovine | Victoria | 1996 | +ve | 6 | + | - | - | +ve, Cattle |
| MAP-147 | Bovine | Victoria | 1993 | +ve | 6 | + | - | - | +ve, Cattle |
| MAP-148 | Bovine | Victoria | 1993 | +ve | 8 | + | - | - | +ve, Cattle |
| MAP-149 | Bovine | Victoria | 1993 | +ve | 8 | + | - | - | +ve, Cattle |
| MAP-150 | Bovine | Victoria | 1993 | +ve | 8 | + | - | - | +ve, Bison |
| MAP-152 | Bovine | Victoria | 1993 | +ve | 8 | + | - | - | +ve, Cattle |
| MAP-153 | Bovine | Victoria | 1993 | +ve | 6 | + | - | - | +ve, Cattle |
| MAP-154 | Bovine | Victoria | 1993 | +ve | 8 | + | - | - | +ve, Cattle |
| MAP-155 | Bovine | Victoria | 1993 | +ve | 6 | + | - | - | +ve, Cattle |
| MAP-156 | Bovine | Victoria | 1993 | +ve | 8 | + | - | - | +ve, Cattle |
| MAP-157 | Bovine | Victoria | 1993 | +ve | 6 | + | - | - | +ve, Cattle |
| MAP-158 | Bovine | Victoria | 1993 | +ve | 8 | + | - | - | +ve, Cattle |
| MAP-159 | Bovine | Victoria | 1995 | +ve | 6 | + | - | - | +ve, Cattle |
| MAP-160 | Human | Victoria | 1993 | +ve | 8 | + | - | - | +ve, Cattle |
| MAP-161 | Bovine | Victoria | 1993 | +ve | 6 | + | - | - | +ve, Cattle |
| MAP-162 | Bovine | Victoria | 1993 | +ve | 8 | + | - | - | +ve, Cattle |
| MAP-163 | Bovine | Victoria | 1993 | +ve | 8 | + | - | - | +ve, Cattle |
| MAP-164 | Bovine | Victoria | 1993 | +ve | 8 | + | - | - | +ve, Cattle |
| MAP-165 | Bovine | Victoria | 1993 | +ve | 8 | + | - | - | +ve, Cattle |
| MAP-166 | Bovine | Victoria | 1996 | +ve | 6 | + | - | - | +ve, Cattle |
| MAP-167 | Bovine | Victoria | 1995 | +ve | 6 | + | - | - | +ve, Cattle |
| MAP-169 | Bovine | Victoria | 1995 | +ve | 8 | + | - | - | +ve, Cattle |
| MAP-170 | Bovine | Victoria | 1996 | +ve | 8 | + | - | - | +ve, Cattle |
| MAP-171 | Bovine | Victoria | 1996 | +ve | 8 | + | - | - | +ve, Cattle |
| MAP-172 | Bovine | Victoria | 1996 | +ve | 1 | + | - | - | +ve, Cattle |
| **Isolate** | **Host** | **Location** | **Year** | **IS900** | **Type C Clade** | **LSP-20** | **LSP-18** | **LSP-4** | **IS1311/REA** |
| MAP-174 | Caprine | France | 1989 | +ve | 1 | + | - | - | +ve, Cattle |
| MAP-175 | Caprine | France | 1989 | +ve | 1 | + | - | - | +ve, Cattle |
| MAP-176 | Caprine | France | 1989 | +ve | 1 | + | - | - | +ve, Cattle |
| MAP-202 | Bovine | Victoria | 1993 | +ve | 6 | + | - | - | +ve, Cattle |
| MAP-203 | Bovine | Victoria | 1993 | +ve | 6 | + | - | - | +ve, Cattle |
| MAP-204 | Bovine | Victoria | 1993 | +ve | 6 | + | - | - | +ve, Cattle |
| MAP-205 | Bovine | Victoria | 1995 | +ve | 6 | + | - | - | +ve, Cattle |
| MAP-206 | Bovine | Victoria | 1996 | +ve | 8 | + | - | - | +ve, Cattle |
| MAP-207 | Bovine | Victoria | 1996 | +ve | 8 | + | - | - | +ve, Cattle |
| MAP-208 | Bovine | Victoria | 1996 | +ve | 1 | + | - | - | +ve, Cattle |
| MAP-209 | Bovine | Victoria | 1996 | +ve | 1 | + | - | - | +ve, Cattle |
| MAP-210 | Bovine | Victoria | 1996 | +ve | 6 | + | - | - | +ve, Cattle |
| MAP-211 | Bovine | Victoria | 1996 | +ve | 8 | + | - | - | +ve, Cattle |
| MAP-212 | Bovine | Victoria | 1996 | +ve | 8 | + | - | - | +ve, Cattle |
| MAP-213 | Bovine | Victoria | 1985 | +ve | 6 | + | - | - | +ve, Cattle |
| MAP-214 | Bovine | Victoria | 1985 | +ve | 6 | + | - | - | +ve, Cattle |
| MAP-215 | Bovine | Victoria | 1985 | +ve | 6 | + | - | - | +ve, Cattle |
| MAP-216 | Bovine | Victoria | - | +ve | 6 | + | - | - | +ve, Cattle |
| MAP-217 | Bovine | France | 1989 | +ve | 1 | + | - | - | +ve, Cattle |
| MAP-218 | Bovine | Victoria | 1993 | +ve | 6 | + | - | - | +ve, Cattle |
| MAP-219 | Bovine | Victoria | 1993 | +ve | 8 | + | - | - | +ve, Cattle |
| MAP-221 | Bovine | Victoria | 1996 | +ve | 6 | + | - | - | +ve, Cattle |
| MAP-222 | Bovine | Victoria | 1996 | +ve | 8 | + | - | - | +ve, Cattle |
| MAP-223 | Bovine | Victoria | 1996 | +ve | 6 | + | - | - | +ve, Cattle |
| MAP-224 | Bovine | Victoria | 1985 | +ve | 6 | + | - | - | +ve, Cattle |
| MAP-225 | Camelid | SA* | 1997 | +ve | 6 | + | - | - | +ve, Cattle |
| MAP-226 | Bovine | Victoria | 1995 | +ve | 8 | + | - | - | +ve, Cattle |
| MAP-227 | Bovine | Victoria | 1997 | +ve | 8 | + | - | - | +ve, Cattle |
| MAP-228 | Bovine | Victoria | 1995 | +ve | 6 | + | - | - | +ve, Cattle |
| MAP-229 | Bovine | Victoria | 1997 | +ve | 8 | + | - | - | +ve, Cattle |
| **Isolate** | **Host** | **Location** | **Year** | **IS900** | **Type C Clade** | **LSP-20** | **LSP-18** | **LSP-4** | **IS1311/REA** |
| MAP-230 | Bovine | Victoria | 1997 | +ve | 8 | + | - | - | +ve, Cattle |
| MAP-233 | Bovine | Victoria | 1997 | +ve | 8 | + | - | - | +ve, Cattle |
| MAP-235 | Bovine | Victoria | 1996 | +ve | 8 | + | - | - | +ve, Cattle |
| MAP-241 | Bovine | Victoria | 1997 | +ve | 8 | + | - | - | +ve, Cattle |
| MAP-242 | Bovine | Victoria | 1997 | +ve | 8 | + | - | - | +ve, Cattle |
| MAP-243 | Bovine | Victoria | 1997 | +ve | 8 | + | - | - | +ve, Cattle |
| MAP-244 | Bovine | Victoria | 1997 | +ve | 8 | + | - | - | +ve, Cattle |
| MAP-245 | Bovine | Victoria | 1997 | +ve | 8 | + | - | - | +ve, Cattle |
| MAP-246 | Bovine | Victoria | 1997 | +ve | 8 | + | - | - | +ve, Cattle |
| MAP-247 | Bovine | Victoria | 1997 | +ve | 8 | + | - | - | +ve, Cattle |
| MAP-249 | Bovine | Victoria | 1997 | +ve | 8 | + | - | - | +ve, Cattle |
| MAP-250 | Bovine | Victoria | 1997 | +ve | 8 | + | - | - | +ve, Cattle |
| MAP-251 | Bovine | Victoria | 1997 | +ve | 8 | + | - | - | +ve, Cattle |
| MAP-252 | Bovine | Victoria | 1997 | +ve | 8 | + | - | - | +ve, Cattle |
| MAP-253 | Bovine | Victoria | 1997 | +ve | 8 | + | - | - | +ve, Cattle |
| MAP-254 | Bovine | Victoria | 1997 | +ve | 8 | + | - | - | +ve, Cattle |
| MAP-271 | Ovine | Victoria | 1999 | +ve | - | - | + | + | +ve, Sheep |
| MAP-273 | Ovine | Victoria | 1997 | +ve | - | - | + | + | +ve, Sheep |
| MAP-274 | Cervine | Victoria | - | +ve | 1 | + | - | - | +ve, Cattle |
| MAP-304 | Bovine | Victoria | 2005 | +ve | 6 | + | - | - | +ve, Cattle |
| MAP-305 | Bovine | Victoria | 2005 | +ve | 6 | + | - | - | +ve, Cattle |
| MAP-306 | Ovine | Victoria | 2005 | +ve | - | - | + | + | +ve, Sheep |
| MAP-320 | Ovine | Victoria | 2005 | +ve | - | - | + | + | +ve, Sheep |
| MAP-322 | Ovine | Victoria | 2005 | +ve | 8 | + | - | - | +ve, Sheep |
| MAP-323 | Ovine | Victoria | 2005 | +ve | - | - | + | + | +ve, Sheep |
| MAP-324 | Ovine | Victoria | 2005 | +ve | - | - | + | + | +ve, Sheep |
| MAP-327 | Ovine | Victoria | 2005 | +ve | - | - | + | + | +ve, Sheep |
| MAP-328 | Ovine | Victoria | 2005 | +ve | - | - | + | + | +ve, Sheep |
| MAP-330 | Ovine | Victoria | 2005 | +ve | - | - | + | + | +ve, Sheep |
| MAP-332 | Ovine | Victoria | 2005 | +ve | - | - | + | + | +ve, Sheep |
| **Isolate** | **Host** | **Location** | **Year** | **IS900** | **Type C Clade** | **LSP-20** | **LSP-18** | **LSP-4** | **IS1311/REA** |
| MAP-333 | Ovine | Victoria | 2005 | +ve | - | - | + | + | +ve, Sheep |
| MAP-339 | Ovine | Victoria | 2005 | +ve | - | - | + | + | +ve, Cattle |
| MAP-340 | Bovine | Victoria | 2005 | +ve | - | - | + | + | +ve, Cattle |
| MAP-341 | Ovine | Victoria | 2005 | +ve | - | - | **+** | **+** | +ve, Sheep |
| MAP-342 | Ovine | Victoria | 2005 | +ve | - | - | **+** | **+** | +ve, Sheep |
| MAP-346 | Ovine | Victoria | 2005 | +ve | - | - | + | + | +ve, Sheep |
| MAP-347 | Ovine | Victoria | 2005 | +ve | - | - | **+** | **+** | +ve, Sheep |
| MAP-348 | Ovine | Victoria | 2005 | +ve | - | - | + | + | +ve, Sheep |
| MAP-349 | Ovine | Victoria | 2005 | +ve | - | - | + | + | +ve, Sheep |
| MAP-350 | Ovine | Victoria | 2005 | +ve | - | + | - | - | +ve, sheep |
| MAP-351 | Ovine | Victoria | 2005 | +ve | - | - | + | + | +ve, Sheep |
| MAP-353 | Bovine | Victoria | 2005 | +ve | 8 | + | - | - | +ve, Cattle |
| MAP-372 | Ovine | Victoria | 2005 | +ve | - | - | + | + | +ve, Sheep |
| MAP-403 | Bovine | Victoria | 2006 | +ve | 8 | + | - | - | +ve, Sheep |
| MAP-404 | Bovine | Victoria | 2006 | +ve | 8 | + | - | - | +ve, Cattle |
| MAP-407 | Bovine | Victoria | 2006 | +ve | 6 | + | - | - | +ve, Cattle |
| MAP-408 | Bovine | Victoria | 2006 | +ve | - | + | - | - | +ve, Cattle |
| MAP-409 | Bovine | Victoria | 2006 | +ve | 8 | + | - | - | +ve, Cattle |
| MAP-411 | Bovine | Victoria | 2006 | +ve | 8 | + | - | - | +ve, Cattle |
| MAP-413 | Bovine | Victoria | 2006 | +ve | 8 | + | - | - | +ve, Cattle |
| MAP-414 | Bovine | Victoria | 2006 | +ve | 8 | + | - | - | +ve, Cattle |
| MAP-415 | Bovine | Victoria | 2006 | +ve | 8 | + | - | - | +ve, Cattle |
| MAP-416 | Bovine | Victoria | 2006 | +ve | - | + | - | - | +ve, Cattle |
| MAP-417 | Bovine | Victoria | 2006 | +ve | 8 | + | - | - | +ve, Cattle |
| MAP-418 | Bovine | Victoria | 2006 | +ve | 8 | + | - | - | +ve, Cattle |
| MAP-419 | Bovine | Victoria | 2006 | +ve | 8 | + | - | - | +ve, Cattle |
| MAP-420 | Bovine | Victoria | 2006 | +ve | 6 | + | - | - | +ve, Cattle |
| MAP-422 | Bovine | Victoria | 2006 | +ve | 8 | + | - | - | +ve, Cattle |
| MAP-423 | Bovine | Victoria | 2006 | +ve | 8 | + | - | - | +ve, Cattle |
| MAP-424 | Bovine | Victoria | 2006 | +ve | 6 | + | - | - | +ve, Cattle |
| **Isolate** | **Host** | **Location** | **Year** | **IS900** | **Type C Clade** | **LSP-20** | **LSP-18** | **LSP-4** | **IS1311/REA** |
| MAP-425 | Bovine | Victoria | 2006 | +ve | 6 | + | - | - | +ve, Cattle |
| MAP-426 | Bovine | Victoria | 2006 | +ve | - | + | - | - | +ve, Cattle |
| MAP-428 | Bovine | Victoria | 2006 | +ve | 8 | + | - | - | +ve, Cattle |
| MAP-429 | Bovine | Victoria | 2006 | +ve | 6 | + | - | - | +ve, Cattle |
| MAP-431 | Bovine | Victoria | 2006 | +ve | 8 | + | - | - | +ve, Cattle |
| MAP-434 | Bovine | Victoria | 2006 | +ve | 8 | + | - | - | +ve, Cattle |
| MAP-435 | Bovine | Victoria | 2006 | +ve | 8 | + | - | - | +ve, Cattle |
| MAP-436 | Bovine | Victoria | 2006 | +ve | 8 | + | - | - | +ve, Cattle |
| MAP-438 | Bovine | Victoria | 2006 | +ve | 6 | + | - | - | +ve, Cattle |
| MAP-439 | Bovine | Victoria | 2006 | +ve | 8 | + | - | - | +ve, Cattle |
| MAP-440 | Bovine | Victoria | 2006 | +ve | - | + | - | - | +ve, Cattle |
| MAP-442 | Bovine | Victoria | 2007 | +ve | 8 | + | - | - | +ve, Cattle |
| MAP-443 | Bovine | Victoria | 2007 | +ve | 8 | + | - | - | +ve, Cattle |
| MAP-444 | Bovine | Victoria | 2007 | +ve | 8 | + | - | - | +ve, Cattle |
| MAP-445 | Bovine | Victoria | 2007 | +ve | 8 | + | - | - | +ve, Cattle |
| MAP-461 | Bovine | Victoria | 2005 | +ve | 6 | + | - | - | +ve, Cattle |
| MAP-506 | Bovine | QLD** | 2015 | +ve | 6 | + | - | - | +ve, Cattle |
| MAP-509 | Bovine | Victoria | 2016 | +ve | 6 | + | - | - | +ve, Cattle |
| MAP-513 | Bovine | QLD | 2016 | +ve | - | + | - | - | +ve, Cattle |
| MAP-514 | Ovine | Victoria | 2016 | +ve | - | - | + | + | +ve, Sheep |
| MAP-515 | Ovine | Victoria | 2016 | +ve | - | - | + | + | +ve, Sheep |
| MAP-517 | Ovine | Victoria | 2015 | +ve | - | - | + | + | +ve, Sheep |
| MAP-520 | Bovine | Victoria | 2015 | +ve | - | + | - | - | +ve, Bison |
| MAP-521 | Bovine | QLD | 2015 | +ve | 6 | + | - | - | +ve, Cattle |
| MAP-524 | Bovine | QLD | 2015 | +ve | - | + | - | - | +ve, Cattle |
| MAP-525 | Bovine | QLD | 2016 | +ve | 8 | + | - | - | +ve, Cattle |
| MAP-526 | Bovine | QLD | 2016 | +ve | 8 | + | - | - | +ve, Cattle |
| MAP-527 | Ovine | QLD | 2016 | +ve | - | - | + | + | +ve, Sheep |
| MAP-529 | Bovine | NSW*** | 2016 | +ve | 8 | + | - | - | +ve, Cattle |
| MAP-533 | Bovine | NSW | 2016 | +ve | 6 | + | - | - | +ve, Cattle |
| **Isolate** | **Host** | **Location** | **Year** | **IS900** | **Type C Clade** | **LSP-20** | **LSP-18** | **LSP-4** | **IS1311/REA** |
| MAP-534 | Bovine | NSW | 2016 | +ve | 6 | + | - | - | +ve, Cattle |
| MAP-535 | Bovine | NSW | 2016 | +ve | 6 | + | - | - | +ve, Cattle |
| MAP-536 | Bovine | NSW | 2016 | +ve | 6 | + | - | - | +ve, Cattle |
| MAP-538 | Bovine | NSW | 2016 | +ve | 6 | + | - | - | +ve, Cattle |
| MAP-539 | Bovine | NSW | 2016 | +ve | 6 | + | - | - | +ve, Cattle |
| MAP-540 | Bovine | NSW | 2016 | +ve | 6 | + | - | - | +ve, Cattle |
| MAP-541 | Bovine | NSW | 2016 | +ve | 6 | + | - | - | +ve, Cattle |
| MAP-551 | Ovine | QLD | 2017 | +ve | - | - | **+** | + | +ve, Sheep |
| MAP-552 | Ovine | QLD | 2017 | +ve | - | - | + | **+** | +ve, Sheep |
| MAP-554 | Bovine | Victoria | 2016 | +ve | - | - | + | + | +ve, Cattle |
| MAP-555 | Ovine | QLD | 2016 | +ve | 6 | + | - | - | +ve, Sheep |
| MAP-556 | Ovine | Victoria | 2016 | +ve | - | - | + | + | +ve, Sheep |
| MAP-557 | Ovine | Victoria | 2016 | +ve | - | - | + | + | +ve, Sheep |
| MAP-558 | Ovine | Victoria | 2017 | +ve | - | - | + | + | +ve, Sheep |
| MAP-559 | Ovine | Victoria | 2017 | +ve | - | - | + | + | +ve, Sheep |
| MAP-560 | Bovine | Victoria | 2017 | +ve | 6 | + | - | - | +ve, Cattle |
| MAP-561 | Ovine | Victoria | 2017 | +ve | - | - | **+** | **+** | +ve, Sheep |
| MAP-562 | Caprine | Victoria | 2017 | +ve | - | - | + | + | +ve, Sheep |
| MAP-564 | Bovine | Victoria | 2017 | +ve | 8 | + | - | - | +ve, Cattle |
| MAP-567 | Bovine | Victoria | 2017 | +ve | 8 | + | - | - | +ve, Cattle |
| MAP-568 | Bovine | Victoria | 2018 | +ve | 8 | + | - | - | +ve, Cattle |
| MAP-570 | Ovine | WA**** | 2018 | +ve | - | - | + | + | +ve, Sheep |
| MAP-571 | Ovine | WA | 2018 | +ve | - | - | + | + | +ve, Sheep |
| MAP-577 | Ovine | WA | 2018 | +ve | - | - | + | + | +ve, Sheep |
| MAP-579 | Ovine | WA | 2018 | +ve | - | - | + | + | +ve, Sheep |
| MAP-580 | Ovine | WA | 2018 | +ve | - | - | + | + | +ve, Sheep |
| MAP-581 | Ovine | WA | 2018 | +ve | - | - | + | + | +ve, Sheep |
| MAP-583 | Ovine | WA | 2018 | +ve | - | - | + | + | +ve, Sheep |
| MAP-584 | Ovine | WA | 2018 | +ve | - | - | + | + | +ve, Sheep |
| MAP-585 | Ovine | WA | 2018 | +ve | - | - | + | + | +ve, Sheep |
| **Isolate** | **Host** | **Location** | **Year** | **IS900** | **Type C Clade** | **LSP-20** | **LSP-18** | **LSP-4** | **IS1311/REA** |
| MAP-586 | Ovine | WA | 2018 | +ve | - | - | + | + | +ve, Sheep |
| MAP-587 | Ovine | WA | 2018 | +ve | - | - | + | + | +ve, Sheep |
| MAP-588 | Ovine | Victoria | 2018 | +ve | - | - | + | + | +ve, Sheep |
| MAP-589 | Ovine | Victoria | 2018 | +ve | - | - | + | + | +ve, Sheep |
| MAP-592 | Ovine | WA | 2018 | +ve | - | - | + | + | +ve, Sheep |
| MAP-594 | Ovine | QLD | 2016 | +ve | - | - | **+** | + | +ve, Sheep |
| MAP-595 | Bovine | Victoria | 2015 | +ve | 8 | + | - | - | +ve, Cattle |
| MAP-596 | Ovine | Victoria | 2015 | +ve | - | + | - | - | +ve, Cattle |
| MAP-597 | Ovine | QLD | 2016 | +ve | - | - | **+** | + | +ve, Sheep |
| MAP-598 | Ovine | QLD | 2016 | +ve | - | - | + | **+** | +ve, Sheep |
| MAP-599 | Bovine | QLD | 2016 | +ve | 6 | + | - | - | +ve, Cattle |
| MAP-602 | Ovine | Victoria | 2017 | +ve | - | - | + | + | +ve, Sheep |
| MAP-607 | Bovine | Victoria | 2018 | +ve | 6 | + | - | - | +ve, Cattle |
| MAP-608 | Bovine | Victoria | 2018 | +ve | 6 | + | - | - | +ve, Cattle |
| MAP-609 | Bovine | Victoria | 2018 | +ve | 8 | + | - | - | +ve, Cattle |
| MAP-610 | Bovine | Victoria | 2018 | +ve | 6 | + | - | - | +ve, Cattle |
| MAP-611 | Bovine | Victoria | 2018 | +ve | 6 | + | - | - | +ve, Cattle |
| MAP-612 | Caprine | Victoria | 2018 | +ve | - | - | + | + | +ve, Cattle |
| MAP-613 | Ovine | WA | 2018 | +ve | - | - | + | + | +ve, Sheep |
| MAP-617 | Ovine | Victoria | 2018 | +ve | - | - | + | + | +ve, Sheep |
| MAP-618 | Bovine | Victoria | 2018 | +ve | 6 | + |  | - | +ve, Cattle |
| MAP-619 | Bovine | Victoria | 2019 | +ve | 8 | + | - | - | +ve, Cattle |
| MAP-621 | Ovine | Victoria | 2018 | +ve | - | - | + | + | +ve, Sheep |
| MAP-623 | Ovine | Victoria | 2018 | +ve | - | - | + | + | +ve, Sheep |

| **Isolate** | **Host** | **Location** | **Year** | **IS900** | **Type C Clade** | **LSP-20** | **LSP-18** | **LSP-4** | **IS1311/REA** |
| --- | --- | --- | --- | --- | --- | --- | --- | --- | --- |
| MAPMRI0102 | Bovine | Italy | 2009 | +ve | - | + | - | + | +ve, Cattle |
| MAPMRI0104 | Bovine | Scotland | 2008 | +ve | 5 | + | - | - | +ve, Cattle |
| MAPMRI0106 | Bovine | Scotland | 2007 | +ve | 1 | + | - | - | +ve, Cattle |
| MAPMRI0107 | Bovine | Scotland | 2008 | +ve | 5 | + | - | - | +ve, Cattle |
| MAPMRI0108 | Bovine | England | 2008 | +ve | 5 | + | - | - | +ve, Cattle |
| MAPMRI0109 | Bovine | Ireland | 2007 | +ve | 1 | + | - | - | +ve, Cattle |
| MAPMRI0111 | Bovine | Scotland | 2008 | +ve | 5 | + | - | - | +ve, Cattle |
| MAPMRI0112 | Bovine | Ireland | 2007 | +ve | 4 | + | - | - | +ve, Cattle |
| MAPMRI0113 | Bovine | Ireland | 2007 | +ve | 4 | + | - | - | +ve, Cattle |
| MAPMRI0114 | Bovine | Ireland | 2007 | +ve | 1 | + | - | - | +ve, Cattle |
| MAPMRI0115 | Ovine | Norway | 2007 | +ve | 5 | **+** | - | - | +ve, Cattle |
| MAPMRI0116 | Bovine | Scotland | 2008 | +ve | 1 | **+** | - | - | +ve, Cattle |
| MAPMRI0117 | Bovine | Czech Republic | 2003 | +ve | - | **+** | - | - | +ve, Bison |
| MAPMRI0118 | Caprine | Norway | 2007 | +ve | 5 | **+** | - | - | +ve, Cattle |
| MAPMRI0119 | Bovine | England | 2008 | +ve | 1 | **+** | - | - | +ve, Cattle |
| MAPMRI0121 | Bovine | England | 2008 | +ve | 5 | **+** | - | - | +ve, Cattle |
| MAPMRI0122 | Bovine | Wales | 2006 | +ve | - | **+** | - | - | +ve, Cattle |
| MAPMRI0123 | Caprine | Norway | 2009 | +ve | 5 | **+** | - | - | +ve, Cattle |
| MAPMRI0124 | Ovine | Scotland | 2008 | +ve | 5 | **+** | - | - | +ve, Cattle |
| MAPMRI0125 | Bovine | Norway | 2010 | +ve | 5 | **+** | - | - | +ve, Cattle |
| MAPMRI0126 | - | England | - | +ve | 5 | **+** | - | - | +ve, Cattle |
| MAPMRI0127 | Bovine | Czech Republic | - | +ve | - | **+** | - | - | +ve, Bison |
| MAPMRI0128 | Bovine | India | 2003 | +ve | - | **+** | - | - | +ve, Bison |
| MAPMRI0129 | Bovine | England | - | +ve | 1 | **+** | - | - | +ve, Cattle |
| MAPMRI0131 | Ovine | Scotland | - | +ve | 5 | **+** | - | - | - |
| MAPMRI0132 | Ovine | Scotland | 2002 | +ve | 5 | **+** | - | - | +ve, Cattle |
| MAPMRI0133 | Ovine | Scotland | 2002 | +ve | 5 | **+** | - | - | - |
| MAPMRI0134 | Bovine | USA | 1990 | +ve | 8 | **+** | - | - | +ve, Cattle |
| **Isolate** | **Host** | **Location** | **Year** | **IS900** | **Type C Clade** | **LSP-20** | **LSP-18** | **LSP-4** | **IS1311/REA** |
| MAPMRI0135 | Bovine | USA | 1990 | +ve | 8 | **+** | - | - | - |
| MAPMRI014 | Human | USA | 1984 | +ve | - | + | - | - | +ve, Cattle |
| MAPMRI022 | Bovine | Argentina | 2003 | +ve | 5 | + | - | - | +ve, Cattle |
| MAPMRI023 | Bovine | Venezuela | 2003 | +ve |  | + | - | - | +ve, Cattle |
| MAPMRI026 | Caprine | India | 2003 | +ve | - | + | - | - | +ve, Cattle |
| MAPMRI027 | Bison | USA | - | +ve | 1 | + | - | - | +ve, Cattle |
| MAPMRI028 | Bison | USA | - | +ve | - | + | - | - | +ve, Cattle |
| MAPMRI029 | Bison | USA | - | +ve | - | + | - | - | +ve, Bison |
| MAPMRI030 | Bison | USA | - | +ve | - | + | - | - | +ve, Cattle |
| MAPMRI031 | Bison | USA | - | +ve | - | + | - | - | +ve, Bison |
| MAPMRI032 | Bovine | Netherland | - | +ve | 4 | + | - | - | +ve, Cattle |
| MAPMRI033 | Ovine | Czech Republic | - | +ve | 1 | + | - | - | +ve, Cattle |
| MAPMRI034 | Ovine | Czech Republic | - | +ve | - | + | - | - | +ve, Bison |
| MAPMRI036 | Ovine | Greece | - | +ve | 5 | + | - | - | +ve, Cattle |
| MAPMRI044 | - | Spain | - | +ve | 8 | + | - | - | +ve, Cattle |
| MAPMRI049 | Ovine | Spain | 2002 | +ve | - | - | + | + | +ve, Sheep |
| MAPMRI050 | Bovine | Spain | 2004 | +ve | 2 | + | - | - | +ve, Cattle |
| MAPMRI051 | Ovine | Spain | 1991 | +ve | - | - | **+** | **+** | +ve, Sheep |
| MAPMRI052 | Bovine | Spain | 2005 | +ve | 1 | + | - | - | +ve, Cattle |
| MAPMRI053 | Bovine | Spain | 2003 | +ve | 1 | + | - | - | +ve, Cattle |
| MAPMRI056 | Bovine | Spain | 2005 | +ve | - | + | - | - | +ve, Cattle |
| MAPMRI058 | Caprine | Spain | 2000 | +ve | - | - | **+** | **+** | +ve, Sheep |
| MAPMRI059 | Bovine | Spain | 2003 | +ve | - | + | - | - | +ve, Cattle |
| MAPMRI060 | Bovine | Germany | 2005 | +ve | 2 | + | - | - | - |
| MAPMRI061 | Bovine | Germany | 2005 | +ve | 1 | + | - | - | - |
| MAPMRI062 | Bovine | Germany | 2005 | +ve | 1 | + | - | - | - |
| MAPMRI063 | Cervine | Germany | 2005 | +ve | - | + | - | - | - |
| MAPMRI064 | Cervine | Germany | 2005 | +ve | - | + | - | - | - |
| MAPMRI065 | Bovine | France | 2003 | +ve | 8 | + | - | - | +ve, Cattle |
| MAPMRI066 | Bovine | France | 2003 | +ve | 1 | + | - | - | +ve, Cattle |
| **Isolate** | **Host** | **Location** | **Year** | **IS900** | **Type C Clade** | **LSP-20** | **LSP-18** | **LSP-4** | **IS1311/REA** |
| MAPMRI067 | Caprine | France | 2004 | +ve | 1 | + | - | - | +ve, Cattle |
| MAPMRI068 | Caprine | France | 2004 | +ve | 1 | + | - | - | +ve, Cattle |
| MAPMRI069 | Bovine | UK | - | +ve | 8 | + | - | - | +ve, Cattle |
| MAPMRI070 | Caprine | Norway | - | +ve | 5 | + | - | - | +ve, Cattle |
| MAPMRI071 | Bovine | N Ireland | 1999/2000 | +ve | - | + | - | - | +ve, Cattle |
| MAPMRI072 | Bovine | N Ireland | 1999/2000 | +ve | 1 | + | - | - | +ve, Cattle |
| MAPMRI073 | Ovine | Netherlands | - | +ve | 4 | + | - | - | +ve, Cattle |
| MAPMRI074 | Bovine | Netherlands | - | +ve | - | + | **-** | **-** | +ve, Sheep |
| MAPMRI075 | Ovine | Scotland | - | +ve | 5 | + | - | - | +ve, Cattle |
| MAPMRI076 | Bovine | Wales | 2008 | +ve | 5 | + | - | - | +ve, Cattle |
| MAPMRI077 | Bovine | Scotland | 2008 | +ve | 8 | + | - | - | +ve, Cattle |
| MAPMRI078 | Bovine | Netherlands | - | +ve | 4 | + | - | - | +ve, Cattle |
| MAPMRI080 | Caprine | Netherlands | - | +ve | 1 | + | - | - | +ve, Cattle |
| MAPMRI081 | Bovine | Netherlands | - | +ve | 4 | + | - | - | +ve, Cattle |
| MAPMRI082 | Caprine | Netherlands | - | +ve | - | + | - | - | +ve, Cattle |
| MAPMRI083 | Cervine | Netherlands | - | +ve | - | - | **+** | **+** | +ve, Sheep |
| MAPMRI084 | Bovine | Wales | 2007 | +ve | - | + | - | - | +ve, Cattle |
| MAPMRI085 | Bovine | England | 2007 | +ve | - | + | **+** | **+** | +ve, Cattle |
| MAPMRI087 | Bovine | England | - | +ve | 5 | + | - | - | +ve, Cattle |
| MAPMRI088 | Ovine | Czech Republic | - | +ve | 1 | + | - | - | +ve, Cattle |
| MAPMRI089 | Bovine | Czech Republic | - | +ve | 1 | + | - | - | +ve, Cattle |
| MAPMRI091 | Caprine | Greece | - | +ve | - | + | - | - | +ve, Cattle |
| MAPMRI090 | Cervine | Argentine | 2003 | +ve | 5 | + | - | - | +ve, Cattle |
| MAPMRI094 | Ovine | Spain | - | +ve | 1 | - | + | + | +ve, Sheep |
| MAPMRI095 | Bovine | Italy | - | +ve | 8 | + | - | - | +ve, Cattle |
| MAPMRI096 | Bovine | Italy | - | +ve | 4 | + | - | - | +ve, Cattle |
| MAPMRI097 | Bovine | Italy | - | +ve | 8 | + | - | - | +ve, Cattle |
| MAPMRI098 | Bovine | England | 2008 | +ve | 8 | + | - | - | +ve, Cattle |
| MAPMRI099 | Bovine | Wales | 2008 | +ve | 4 | + | - | - | +ve, Cattle |
| MAPMRI100 | Bovine | Italy | - | +ve | 3 | + | - | - | +ve, Cattle |
| **Isolate** | **Host** | **Location** | **Year** | **IS900** | **Type C Clade** | **LSP-20** | **LSP-18** | **LSP-4** | **IS1311/REA** |
| MAPMRI101 | Bovine | Italy | - | +ve | 4 | + | - | - | +ve, Cattle |
| MAPMRI0103 | Ovine | Scotland | 2004 | +ve | 8 | + | - | - | +ve, Cattle |
| MAPMRI155 | Bovine | USA | 1990 | +ve | 5 | + | - | - | +ve, Cattle |
| MAPMRI156 | Ovine | Scotland | - | +ve | 5 | + | - | - | +ve, Cattle |
| MAPMRI157 | Ovine | Scotland | 2002 | +ve | 8 | + | - | - | +ve, Cattle |
| MAPMRI158 | Bovine | New Zealand | 2010 | +ve | 8 | + | - | - | +ve, Cattle |
| MAPMRI159 | Bovine | New Zealand | 2010 | +ve | 1 | + | - | - | +ve, Cattle |
| MAPMRI160 | Cervine | New Zealand | 2009 | +ve | 2 | + | - | - | +ve, Cattle |
| MAPMRI035 | Ovine | Greece | - | +ve | - | - | + | + | +ve, Sheep |
| SRR1793679 | Bovine | Canada | 1999-2002 | +ve | 6 | + | - | - | +ve, Cattle |
| SRR1793681 | Bovine | Canada | 1999-2002 | +ve | - | + | - | - | +ve, Cattle |
| SRR1793683 | Bovine | Canada | 1999-2002 | +ve | 8 | + | - | - | +ve, Cattle |
| SRR1793685 | Bovine | Canada | 1999-2002 | +ve | - | + | - | - | +ve, Cattle |
| SRR1793698 | Bovine | Canada | 1999-2002 | +ve | - | + | - | - | +ve, Cattle |
| SRR1793700 | Bovine | Canada | 1999-2002 | +ve | 7 | + | - | - | +ve, Cattle |
| SRR1793701 | Bovine | Canada | 1999-2002 | +ve | - | + | - | - | +ve, Cattle |
| SRR1793702 | Bovine | Canada | 1999-2002 | +ve | 8 | + | - | - | +ve, Cattle |
| SRR1793704 | Bovine | Canada | 1999-2002 | +ve | 8 | + | - | - | +ve, Cattle |
| SRR1793703 | Bovine | Canada | 1999-2002 | +ve | 7 | + | - | - | +ve, Cattle |
| SRR1793706 | Bovine | Canada | 1999-2002 | +ve | 8 | + | - | - | +ve, Cattle |
| SRR1793707 | Bovine | Canada | 1999-2002 | +ve | 1 | + | - | - | +ve, Cattle |
| SRR1793708 | Bovine | Canada | 1999-2002 | +ve | 8 | + | - | - | +ve, Cattle |
| SRR1793709 | Bovine | Canada | 1999-2002 | +ve | - | + | - | - | +ve, Cattle |
| SRR1793716 | Bovine | Canada | 1999-2002 | +ve | 3 | + | - | - | +ve, Cattle |
| SRR1793718 | Bovine | Canada | 1999-2002 | +ve | 7 | + | - | - | +ve, Cattle |
| SRR1793719 | Bovine | Canada | 1999-2002 | +ve | - | + | - | - | +ve, Cattle |
| SRR1793720 | Bovine | Canada | 1999-2002 | +ve | 8 | + | - | - | +ve, Cattle |
| SRR1793723 | Bovine | Canada | 1999-2002 | +ve | 8 | + | - | - | +ve, Cattle |
| SRR1793726 | Bovine | Canada | 1999-2002 | +ve | 8 | + | - | - | +ve, Cattle |
| DT3 | Cervine | - | - | +ve | 8 | + | - | - | +ve, Cattle |
| **Isolate** | **Host** | **Location** | **Year** | **IS900** | **Type C Clade** | **LSP-20** | **LSP-18** | **LSP-4** | **IS1311/REA** |
| E93 | Bovine | Egypt | 2011 | +ve | 8 | + | - | - | +ve, Cattle |
| MAP-4 | Human | - | 2014 | +ve | 3 | + | - | - | +ve, Cattle |
| Tn-India | Bison | India | - | +ve | - | + | - | - | +ve, Bison |
| E1 | Bovine | Egypt | 2011 | +ve | - | + | - | - | +ve, Cattle |
| CLIJ644-CSIRO | Bovine | Australia | - | +ve | 6 | + | - | - | +ve, Cattle |

+ve = positive PCR result. *South Australia, **Queensland, ***New South Wales, ****Western Australia

Availability of supporting data section

Raw sequence data for the international isolates can be obtained from the European Nucleotide Archive (ENA) under accession PRJEB2204 and NCBI Bioproject, accession number PRJNA274491.
