# Supplementary figures and images for: Molecular characterisation of Mycobacterium avium subsp. paratuberculosis in Australia
Source: BMC Microbiol. 2021 Apr 1;21:101. doi: 10.1186/s12866-021-02140-2 (PMC8012159; doi:10.1186/s12866-021-02140-2)

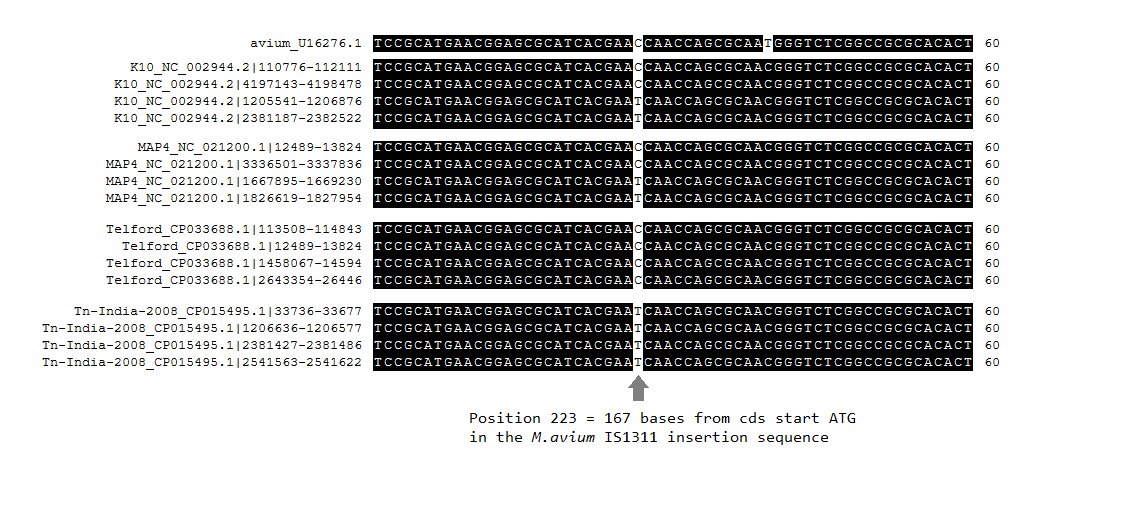

Supplement: Supplementary file 2 — Additional file 2 : Figure S1. Muscle alignment of the IS1311 insertion from M. avium subsp. avium (U16276.1), Map K10 (NC_002944.2), MAP 4 (NC_021200.1), and Telford (CP033688.1) that contains the SNP at position 223 targeted by the restriction enzyme analysis (REA). [file 12866_2021_2140_MOESM2_ESM.png]
