# Supplementary material for: Molecular characterisation of Mycobacterium avium subsp. paratuberculosis in Australia
Source: BMC Microbiol. 2021 Apr 1;21:101. doi: 10.1186/s12866-021-02140-2 (PMC8012159; doi:10.1186/s12866-021-02140-2)
Supplement: Supplementary file 3 — Additional file 3 : Table S2. Australian and International Map isolates that showed cross species transmission. [file 12866_2021_2140_MOESM3_ESM.docx]

**Additional file 3: Table S2.** Australian and International Map isolates that showed

cross species transmission

| **Isolate** | **Host** | **Location** | **IS900** | **IS1311/REA** |
| --- | --- | --- | --- | --- |
| MAP-107 | Bovine | Victoria | +ve | +ve, Avium |
| MAP-115 | Bovine | Victoria | +ve | +ve, Avium |
| MAP-119 | Bovine | Victoria | +ve | +ve, Avium |
| MAP-129 | Ovine | France | +ve | +ve, Cattle |
| MAP-339 | Ovine | Victoria | +ve | +ve, Cattle |
| MAP-340 | Ovine | Victoria | +ve | +ve, Cattle |
| MAP-403 | Bovine | Victoria | +ve | +ve, Sheep |
| MAP-596 | Ovine | Victoria | +ve | +ve, Cattle |
| MAPMRI033 | Ovine | Czech Republic | +ve | +ve, Cattle |
| MAPMRI036 | Ovine | Greece | +ve | +ve, Cattle |
| MAPMRI073 | Ovine | Netherlands | +ve | +ve, Cattle |
| MAPMRI075 | Ovine | Scotland | +ve | +ve, Cattle |
| MAPMRI088 | Ovine | Czech Republic | +ve | +ve, Cattle |
| MAPMRI0103 | Ovine | Scotland | +ve | +ve, Cattle |
| MAPMRI0115 | Ovine | Norway | +ve | +ve, Cattle |
| MAPMRI0132 | Ovine | Scotland | +ve | +ve, Cattle |
| MAPMRI156 | Ovine | Scotland | +ve | +ve, Cattle |
| MAPMRI157 | Ovine | Scotland | +ve | +ve, Cattle |

+ve = positive PCR result
