# Supplementary material for: Molecular characterisation of Mycobacterium avium subsp. paratuberculosis in Australia
Source: BMC Microbiol. 2021 Apr 1;21:101. doi: 10.1186/s12866-021-02140-2 (PMC8012159; doi:10.1186/s12866-021-02140-2)
Supplement: Supplementary file 4 — Additional file 4: Table S3. Sequencing depth and coverage, number of SNP’s before and after filtering when aligned to Map K10 and Telford strain, GC content, number of contigs and number of raw sequence reads generated from the next generation sequencing of isolates that were sequenced and used for phylogenetic analysis in this study against the K10 reference strain [file 12866_2021_2140_MOESM4_ESM.docx]

**Additional File 4: Table S3.** Genomic data for all isolates included in this study including number of raw sequence reads generated, sequence depth, coverage to Map K10, number of SNPs when compared to the K10 reference strain and Telford strain (before and after filtering) and assembly statistics including number of contigs, N50, GC content and total length of assembled genome.

| **Isolate** | **Strain Type** | **No. of raw reads** | **Depth** | **Coverage %** | **No. of SNP’s when aligned to Map K10 before filtering** | **No. of SNP’s when aligned to Map K10 after filtering** | **No. of SNP’s when aligned to Map Telford strain before filtering** | **No. of SNP’s when aligned to Map Telford strain after filtering** | **GC Content** | **No. of Contigs** | **N50** | **Total Length of genome** |
| --- | --- | --- | --- | --- | --- | --- | --- | --- | --- | --- | --- | --- |
| MAP-102 | Cattle | 1614608 | 69 | 99 | 97 | 34 | -* | - | 69.34 | 108 | 91959 | 4742305 |
| MAP-106 | Cattle | 2596732 | 110 | 99 | 99 | 46 | - | - | 69.34 | 98 | 118162 | 4749576 |
| MAP-107 | Avium | 1468604 | 55 | 99 | 15158 | 6253 | - | - | 69.34 | 133 | 87558 | 4931418 |
| MAP-108 | Avium | 1141528 | 44 | 94 | 44846 | 14716 | - | - | 69.32 | 101 | 101720 | 4851396 |
| MAP-112 | Cattle | 2769148 | 122 | 99 | 97 | 35 | - | - | 69.34 | 99 | 112785 | 4748377 |
| MAP-113 | Cattle | 2939092 | 124 | 99 | 130 | 66 | - | - | 69.34 | 101 | 94430 | 4750122 |
| MAP-114 | Cattle | 2329544 | 97 | 99 | 94 | 36 | - | - | 69.34 | 99 | 98025 | 4749422 |
| MAP-115 | Avium | 3168128 | 123 | 99 | 422264 | 14209 | - | - | 69.25 | 110 | 124826 | 4959187 |
| MAP-116 | - | 2211702 | 51 | 99 | 22370 | 4563 | - | - | 67.01 | 130 | 138553 | 5610420 |
| MAP-118 | Cattle | 2603872 | 115 | 99 | 144 | 62 | - | - | 69.34 | 95 | 97190 | 4756943 |
| MAP-119 | Avium | 1508744 | 88 | 96 | 38499 | 14164 | - | - | 69.22 | 133 | 93379 | 4938495 |
| MAP-120 | Cattle | 2094898 | 96 | 99 | 146 | 61 | - | - | 69.34 | 98 | 98150 | 4723214 |
| MAP-121 | Cattle | 3116122 | 142 | 99 | 230 | 85 | - | - | 69.34 | 99 | 92051 | 4746828 |
| MAP-122 | Cattle | 2773260 | 92 | 99 | 143 | 59 | - | - | 69.33 | 117 | 80713 | 4747347 |
| MAP-123 | Cattle | 3260060 | 142 | 99 | 97 | 39 | - | - | 69.34 | 98 | 98027 | 4750449 |
| MAP-125 | Cattle | 2217086 | 77 | 99 | 96 | 37 | - | - | 69.32 | 115 | 80726 | 4741908 |
| MAP-127 | Cattle | 858944 | 35 | 99 | 93 | 35 | - | - | 69.33 | 118 | 86177 | 4742455 |
| MAP-128 | Cattle | 1284826 | 39 | 99 | 132 | 62 | - | - | 69.3 | 162 | 57762 | 4730527 |
| MAP-129 | Cattle | 1667830 | 71 | 99 | 62 | 25 | - | - | 69.34 | 100 | 95590 | 4723737 |
| MAP-131 | Cattle | 1671168 | 72 | 99 | 137 | 59 | - | - | 69.33 | 103 | 91039 | 4731700 |
| MAP-132 | Cattle | 1161190 | 35 | 99 | 80 | 39 | - | - | 69.33 | 129 | 83182 | 4747100 |
| MAP-134 | Cattle | 2689414 | 91 | 99 | 133 | 56 | - | - | 69.34 | 106 | 91972 | 4752676 |
| MAP-135 | Cattle | 2442224 | 80 | 99 | 128 | 60 | - | - | 69.33 | 112 | 91669 | 4750459 |
| MAP-138 | Cattle | 957338 | 45 | 99 | 99 | **35** | - | - | 69.31 | **127** | 73111 | 4671948 |
| MAP-139 | Cattle | 2580538 | 118 | 99 | 97 | 41 | - | - | 69.33 | 100 | 91968 | 4746093 |
| MAP-140 | Cattle | 1279406 | 56 | 99 | 143 | 58 | - | - | 69.34 | 110 | 92226 | 4750597 |
| MAP-141 | Cattle | 1416688 | 62 | 99 | 142 | 58 | - | - | 69.34 | 103 | 92226 | 4750909 |
| MAP-142 | Cattle | 1235156 | 51 | 99 | 145 | 58 | - | - | 69.35 | 109 | 91972 | 4726865 |
| MAP-143 | Cattle | 2602310 | 85 | 99 | 210 | 102 | - | - | 69.33 | 118 | 86178 | 4747858 |
| MAP-144 | Cattle | 2998666 | 141 | 99 | 142 | 58 | - | - | 69.35 | 98 | 92226 | 4704917 |
| MAP-147 | Cattle | 821444 | 33 | 99 | 132 | 55 | - | - | 69.33 | 129 | 74421 | 4742714 |
| MAP-148 | Cattle | 1958504 | 90 | 99 | 90 | 41 | - | - | 69.33 | 113 | 91971 | 4740835 |
| MAP-149 | Cattle | 1594070 | 52 | 99 | 98 | 41 | - | - | 69.32 | 116 | 86179 | 4742037 |
| MAP-150 | Bison | 871516 | 36 | 99 | 87 | 33 | - | - | 69.32 | 117 | 86187 | 4740437 |
| MAP-152 | Cattle | 2941452 | 121 | 99 | 100 | 39 | - | - | 69.34 | 96 | 98039 | 4751363 |
| MAP-153 | Cattle | 2462384 | 107 | 99 | 133 | 60 | - | - | 69.34 | 102 | 97181 | 4733545 |
| MAP-154 | Cattle | 1367406 | 43 | 99 | 86 | 36 | - | - | 69.32 | 115 | 80168 | 4745199 |
| MAP-155 | Cattle | 1920118 | 80 | 99 | 140 | 58 | - | - | 69.34 | 111 | 91972 | 4749965 |
| MAP-156 | Cattle | 1102926 | 33 | 99 | 81 | 38 | - | - | 69.31 | 122 | 76999 | 4738790 |
| MAP-157 | Cattle | 1457448 | 52 | 99 | 140 | 65 | - | - | 69.34 | 108 | 97149 | 4753245 |
| MAP-158 | Cattle | 2166384 | 87 | 99 | 93 | 40 | - | - | 69.34 | 108 | 100117 | 4670073 |
| MAP-159 | Cattle | 872496 | 37 | 99 | 141 | 59 | - | - | 69.34 | 127 | 74810 | 4723807 |
| MAP-160 | Cattle | 2091164 | 75 | 99 | 90 | 40 | - | - | 69.33 | 121 | 91959 | 4721010 |
| MAP-161 | Cattle | 2696800 | 95 | 99 | 127 | 62 | - | - | 69.34 | 102 | 92260 | 4711787 |
| MAP-162 | Cattle | 2073216 | 87 | 99 | 93 | 37 | - | - | 69.34 | 100 | 97218 | 4751818 |
| MAP-163 | Cattle | 1143250 | 43 | 99 | 88 | 38 | - | - | 69.33 | 127 | 86178 | 4744288 |
| MAP-164 | Cattle | 1084104 | 36 | 99 | 80 | 35 | - | - | 69.34 | 126 | 79582 | 4731884 |
| MAP-165 | Cattle | 3063228 | 128 | 99 | 59 | 26 | - | - | 69.37 | 101 | 91865 | 4678078 |
| MAP-166 | Cattle | 957510 | 31 | 99 | 131 | 59 | - | - | 69.35 | 109 | 92226 | 4709794 |
| MAP-167 | Cattle | 1187794 | 50 | 99 | 141 | 58 | - | - | 69.33 | 121 | 91041 | 4752298 |
| MAP-169 | Cattle | 2175030 | 93 | 99 | 97 | 38 | - | - | 69.34 | 105 | 97202 | 4751296 |
| MAP-170 | Cattle | 2816600 | 101 | 99 | 88 | 38 | - | - | 69.34 | 102 | 92203 | 4695002 |
| MAP-171 | Cattle | 3076996 | 137 | 99 | 97 | 39 | - | - | 69.34 | 99 | 91955 | 4748551 |
| MAP-172 | Cattle | 1899430 | 84 | 99 | 231 | 101 | - | - | 69.34 | 99 | 92265 | 4751973 |
| MAP-174 | Cattle | 2991758 | 135 | 99 | 240 | **95** | - | - | 69.34 | **91** | 98033 | 4755118 |
| MAP-175 | Cattle | 2922674 | 93 | 99 | 211 | 97 | - | - | 69.33 | 117 | 85694 | 4744754 |
| MAP-176 | Cattle | 1752578 | 57 | 99 | 243 | 108 | - | - | 69.34 | 108 | 91997 | 4749363 |
| MAP-202 | Cattle | 1270714 | 39 | 99 | 130 | 59 | - | - | 69.29 | 167 | 56039 | 4735369 |
| MAP-203 | Cattle | 3356562 | 147 | 99 | 159 | 59 | - | - | 69.35 | 100 | 97200 | 4740665 |
| MAP-204 | Cattle | 3866158 | 174 | 99 | 150 | 65 | - | - | 69.34 | 91 | 106164 | 4759721 |
| MAP-205 | Cattle | 1363398 | 45 | 99 | 142 | 58 | - | - | 69.33 | 118 | 80163 | 4753536 |
| MAP-206 | Cattle | 1560584 | 50 | 99 | 92 | 38 | - | - | 69.31 | 126 | 83183 | 4739416 |
| MAP-207 | Cattle | 1367520 | 44 | 99 | 84 | 38 | - | - | 69.32 | 126 | 80187 | 4743230 |
| MAP-208 | Cattle | 1500316 | 48 | 99 | 207 | 101 | - | - | 69.33 | 129 | 82783 | 4754090 |
| MAP-209 | Cattle | 1406756 | 44 | 99 | 215 | 101 | - | - | 69.33 | 116 | 85671 | 4748241 |
| MAP-210 | Cattle | 1563926 | 49 | 99 | 137 | 59 | - | - | 69.33 | 110 | 98574 | 4747706 |
| MAP-211 | Cattle | 1243432 | 41 | 99 | 88 | 40 | - | - | 69.31 | 130 | 80169 | 4740288 |
| MAP-212 | Cattle | 1171746 | 37 | 99 | 85 | 37 | - | - | 69.32 | 123 | 88915 | 4740903 |
| MAP-213 | Cattle | 1390258 | 45 | 99 | 98 | 62 | - | - | 69.33 | 114 | 86177 | 4749360 |
| MAP-214 | Cattle | 1444128 | 45 | 99 | 138 | 58 | - | - | 69.31 | 137 | 67807 | 4740573 |
| MAP-215 | Cattle | 1337254 | 43 | 99 | 119 | 56 | - | - | 69.3 | 150 | 65155 | 4698158 |
| MAP-216 | Cattle | 1290216 | 43 | 99 | 141 | 59 | - | - | 69.29 | 159 | 56039 | 4737573 |
| MAP-217 | Cattle | 1362244 | 45 | 99 | 130 | 98 | - | - | 69.32 | 132 | 74332 | 4744905 |
| MAP-218 | Cattle | 1081958 | 34 | 99 | 124 | 59 | - | - | 69.31 | 145 | 69208 | 4741412 |
| MAP-219 | Cattle | 1211672 | 40 | 99 | 82 | 33 | - | - | 69.32 | 122 | 74353 | 4742774 |
| MAP-221 | Cattle | 1322840 | 44 | 99 | 128 | 51 | - | - | 69.32 | 135 | 69216 | 4745117 |
| MAP-222 | Cattle | 1315244 | 43 | 99 | 85 | 37 | - | - | 69.33 | 113 | 91959 | 4744684 |
| MAP-223 | Cattle | 1445104 | 40 | 99 | 3124 | 59 | - | - | 69.34 | 103 | 95252 | 4758487 |
| MAP-224 | Cattle | 1455320 | 47 | 99 | 133 | 62 | - | - | 69.34 | 109 | 91668 | 4756166 |
| MAP-225 | Cattle | 1308034 | 42 | 99 | 140 | 61 | - | - | 69.34 | 107 | 91933 | 4749188 |
| MAP-226 | Cattle | 1668390 | 55 | 99 | 90 | 38 | - | - | 69.32 | 117 | 86177 | 4738021 |
| MAP-227 | Cattle | 1302742 | 41 | 99 | 91 | 40 | - | - | 69.32 | 123 | 84187 | 4740021 |
| MAP-228 | Cattle | 1460248 | 47 | 99 | 129 | 59 | - | - | 69.33 | 108 | 86178 | 4751074 |
| MAP-229 | Cattle | 1092324 | 35 | 99 | 92 | 40 | - | - | 69.32 | 114 | 83035 | 4741746 |
| MAP-230 | Cattle | 1302594 | 42 | 99 | 99 | 39 | - | - | 69.31 | 138 | 74421 | 4742502 |
| MAP-233 | Cattle | 1991316 | 62 | 99 | 91 | 42 | - | - | 69.32 | 113 | 86177 | 4724246 |
| MAP-235 | Cattle | 1541644 | 51 | 99 | 94 | 42 | - | - | 69.31 | 149 | 74421 | 4735210 |
| MAP-241 | Cattle | 1721182 | 56 | 99 | 95 | 41 | - | - | 69.33 | 114 | 91665 | 4746102 |
| MAP-242 | Cattle | 1351332 | 44 | 99 | 100 | 41 | - | - | 69.32 | 117 | 83528 | 4741763 |
| MAP-243 | Cattle | 1259450 | 40 | 99 | 95 | 41 | - | - | 69.32 | 134 | 74421 | 4724832 |
| MAP-244 | Cattle | 1468616 | 48 | 99 | 98 | 40 | - | - | 69.32 | 133 | 76761 | 4739703 |
| MAP-245 | Cattle | 1393398 | 45 | 99 | 97 | 40 | - | - | 69.3 | 135 | 65015 | 4719082 |
| MAP-246 | Cattle | 1719044 | 55 | 99 | 99 | 40 | - | - | 69.33 | 111 | 84174 | 4743944 |
| MAP-247 | Cattle | 1624094 | 53 | 99 | 93 | 38 | - | - | 69.33 | 112 | 84159 | 4742517 |
| MAP-249 | Cattle | 1348290 | 45 | 99 | 102 | 40 | - | - | 69.33 | 114 | 86177 | 4743995 |
| MAP-250 | Cattle | 1103698 | 36 | 99 | 90 | 40 | - | - | 69.29 | 160 | 58009 | 4726574 |
| MAP-251 | Cattle | 1191372 | 37 | 99 | 94 | 39 | - | - | 69.3 | 138 | 74336 | 4738100 |
| MAP-252 | Cattle | 1294336 | 43 | 99 | 92 | 40 | - | - | 69.31 | 132 | 69259 | 4737052 |
| MAP-253 | Cattle | 1324070 | 44 | 99 | 95 | 39 | - | - | 69.32 | 128 | 69259 | 4737487 |
| MAP-254 | Cattle | 1205108 | 38 | 99 | 144 | 41 | - | - | 69.32 | 124 | 80730 | 4743528 |
| MAP-271 | Sheep | 1465070 | 48 | 99 | 3347 | 1507 | 38 | 22 | 69.27 | 159 | 58770 | 4775688 |
| MAP-273 | Sheep | 1560782 | 51 | 99 | 2311 | 1512 | 37 | 21 | 69.23 | 204 | 47229 | 4781990 |
| MAP-274 | Cattle | 1105674 | 36 | 99 | 220 | 108 | - | - | 69.3 | 161 | 65061 | 4740085 |
| MAP-304 | Cattle | 1348196 | 44 | 99 | 123 | 56 | - | - | 69.32 | 136 | 80141 | 4749050 |
| MAP-305 | Cattle | 1335884 | 44 | 99 | 128 | 57 | - | - | 69.31 | 131 | 74351 | 4744388 |
| MAP-306 | Sheep | 1369454 | 44 | 99 | 3219 | 1509 | 24 | 12 | 69.28 | 143 | 72147 | 4796317 |
| MAP-320 | Sheep | 1309528 | 42 | 99 | 3202 | 1506 | 260 | 139 | 69.27 | 155 | 69117 | 4765203 |
| MAP-322 | Sheep | 895496 | 30 | 99 | 100 | 40 | 3089 | 1525 | 69.25 | 203 | 44844 | 4713209 |
| MAP-323 | Sheep | 1233954 | 41 | 99 | 3149 | 1508 | 250 | 139 | 69.26 | 172 | 56124 | 4789954 |
| MAP-324 | Sheep | 1145918 | 35 | 99 | 2999 | 1509 | 11 | 9 | 69.26 | 169 | 52217 | 4789162 |
| MAP-327 | Sheep | 1088568 | 32 | 99 | 2856 | 1513 | 21 | 11 | 69.26 | 163 | 61823 | 4790375 |
| MAP-328 | Sheep | 1235898 | 39 | 99 | 3243 | 1512 | 24 | 14 | 69.27 | 154 | 74302 | 4794394 |
| MAP-330 | Sheep | 1299586 | 42 | 98 | 3259 | 1508 | 36 | 22 | 69.21 | 230 | 40699 | 4765203 |
| MAP-332 | Sheep | 1247934 | 41 | 99 | 3282 | 1501 | 36 | 16 | 69.28 | 146 | 74302 | 4800622 |
| MAP-333 | Sheep | 1170396 | 38 | 99 | 3238 | 1506 | 34 | 16 | 69.25 | 200 | 48086 | 4788831 |
| MAP-339 | Cattle | 1002104 | 32 | 99 | 3141 | 1506 | 12 | 20 | 69.23 | 193 | 50067 | 4756988 |
| MAP-340 | Cattle | 1762858 | 55 | 99 | 3193 | 1511 | 17 | 10 | 69.24 | 182 | 57608 | 4785595 |
| MAP-341 | Sheep | 1821810 | 57 | 99 | 3242 | 1513 | 18 | 12 | 69.26 | 175 | 52458 | 4789402 |
| MAP-342 | Sheep | 1492988 | 49 | 99 | 3277 | 1511 | 16 | 10 | 69.27 | 156 | 61823 | 4795820 |
| MAP-346 | Sheep | 1314434 | 43 | 99 | 3284 | 1502 | 38 | 16 | 69.18 | 245 | 37567 | 4755433 |
| MAP-347 | Sheep | 1265832 | 41 | 99 | 3250 | 1503 | 38 | 16 | 69.26 | 167 | 59627 | 4790452 |
| MAP-348 | Sheep | 1603044 | 54 | 99 | 3373 | 1509 | 25 | 14 | 69.24 | 208 | 44785 | 4768832 |
| MAP-349 | Sheep | 1452996 | 47 | 99 | 3287 | 1511 | 48 | 29 | 69.27 | 157 | 61820 | 4794104 |
| MAP-350 | Sheep | 1096088 | 35 | 99 | 57 | 33 | 2897 | 1498 | 69.29 | 180 | 51237 | 4685992 |
| MAP-351 | Sheep | 1686328 | 54 | 99 | 3332 | 1518 | 21 | 15 | 69.28 | 142 | 80160 | 4800641 |
| MAP-353 | Cattle | 1236846 | 39 | 99 | 80 | 35 | - | - | 69.32 | 124 | 88900 | 4738729 |
| MAP-372 | Sheep | 897960 | 29 | 99 | 3135 | 1505 | 241 | 139 | 69.25 | 184 | 50075 | 4769094 |
| MAP-403 | Sheep | 1049718 | 31 | 99 | 83 | 42 | 2716 | 1526 | 69.31 | 127 | 76999 | 4738691 |
| MAP-404 | Cattle | 1311344 | 44 | 99 | 91 | 36 | - | - | 69.31 | 132 | 72959 | 4734879 |
| MAP-407 | Cattle | 1633642 | 53 | 99 | 135 | 59 | - | - | 69.31 | 158 | 56041 | 4680742 |
| MAP-408 | Cattle | 1428244 | 46 | 99 | 104 | 53 | - | - | 69.31 | 139 | 59400 | 4738561 |
| MAP-409 | Cattle | 1456064 | 46 | 99 | 90 | 34 | - | - | 69.33 | 141 | 69259 | 4686670 |
| MAP-411 | Cattle | 977038 | 33 | 99 | 91 | 39 | - | - | 69.28 | 182 | 55842 | 4722812 |
| MAP-413 | Cattle | 1403252 | 46 | 99 | 91 | 36 | - | - | 69.31 | 132 | 74323 | 4732586 |
| MAP-414 | Cattle | 1108454 | 35 | 99 | 92 | 40 | - | - | 69.31 | 131 | 69451 | 4739720 |
| MAP-415 | Cattle | 1364816 | 43 | 99 | 88 | 41 | - | - | 69.31 | 137 | 76999 | 4734676 |
| MAP-416 | Cattle | 1291666 | 40 | 99 | 115 | 52 | - | - | 69.31 | 138 | 69447 | 4739818 |
| MAP-417 | Cattle | 1545252 | 50 | 99 | 93 | 42 | - | - | 69.31 | 123 | 80732 | 4718781 |
| MAP-418 | Cattle | 1288336 | 39 | 99 | 55 | 25 | - | - | 69.27 | 172 | 53661 | 4724444 |
| MAP-419 | Cattle | 1497186 | 50 | 99 | 97 | 42 | - | - | 69.31 | 143 | 74381 | 4735627 |
| MAP-420 | Cattle | 1273058 | 41 | 99 | 135 | 62 | - | - | 69.31 | 135 | 69216 | 4742971 |
| MAP-422 | Cattle | 1441958 | 45 | 99 | 94 | 34 | 2947 | 1523 | 69.33 | 112 | 91923 | 4739792 |
| MAP-423 | Cattle | 1334988 | 44 | 99 | 93 | 38 | - | - | 69.32 | 121 | 80153 | 4740597 |
| MAP-424 | Cattle | 1306878 | 42 | 99 | 120 | 65 | - | - | 69.32 | 120 | 91668 | 4750273 |
| MAP-425 | Cattle | 1416702 | 46 | 99 | 130 | 59 | - | - | 69.32 | 123 | 83179 | 4733549 |
| MAP-426 | Cattle | 997048 | 31 | 99 | 104 | 52 | - | - | 69.31 | 155 | 58011 | 4735515 |
| MAP-428 | Cattle | 1105912 | 34 | 99 | 88 | 45 | - | - | 69.28 | 171 | 52509 | 4723597 |
| MAP-429 | Cattle | 1112718 | 35 | 99 | 123 | 57 | - | - | 69.31 | 147 | 59379 | 4736354 |
| MAP-431 | Cattle | 1024742 | 31 | 99 | 85 | 43 | - | - | 69.31 | 127 | 78937 | 4737062 |
| MAP-434 | Cattle | 1004788 | 32 | 99 | 90 | 42 | - | - | 69.3 | 141 | 58963 | 4732302 |
| MAP-435 | Cattle | 1070212 | 34 | 99 | 88 | 39 | - | - | 69.3 | 144 | 58009 | 4730477 |
| MAP-436 | Cattle | 1974936 | 63 | 99 | 89 | 44 | - | - | 69.29 | 158 | 58500 | 4726109 |
| MAP-438 | Cattle | 998262 | 31 | 99 | 130 | 60 | - | - | 69.32 | 125 | 80697 | 4743088 |
| MAP-439 | Cattle | 1050614 | 33 | 99 | 91 | 42 | - | - | 69.31 | 129 | 80153 | 4737591 |
| MAP-440 | Cattle | 1706516 | 53 | 99 | 65 | 32 | - | - | 69.34 | 117 | 86177 | 4740554 |
| MAP-442 | Cattle | 1209600 | 37 | 99 | 71 | 32 | - | - | 69.33 | 144 | 74367 | 4683256 |
| MAP-443 | Cattle | 1037646 | 33 | 99 | 95 | 38 | - | - | 69.31 | 129 | 69259 | 4739181 |
| MAP-444 | Cattle | 1084026 | 34 | 99 | 92 | 41 | - | - | 69.29 | 154 | 65030 | 4721288 |
| MAP-445 | Cattle | 1029118 | 33 | 99 | 88 | 41 | - | - | 69.31 | 128 | 76808 | 4739126 |
| MAP-461 | Cattle | 1894688 | 64 | 99 | 138 | 60 | - | - | 69.32 | 118 | 80168 | 4750824 |
| MAP-506 | Cattle | 972030 | 31 | 99 | 124 | 58 | - | - | 69.31 | 138 | 65012 | 4740561 |
| MAP-509 | Cattle | 1496680 | 52 | 99 | 142 | 60 | - | - | 69.32 | 121 | 74421 | 4651437 |
| MAP-513 | Cattle | 1332182 | 54 | 99 | 127 | 56 | - | - | 69.33 | 119 | 86177 | 4747183 |
| MAP-514 | Sheep | 1320434 | 43 | 99 | 3288 | 1057 | 19 | 10 | 69.24 | 186 | 51692 | 4784025 |
| MAP-515 | Sheep | 1724184 | 57 | 99 | 3346 | 1510 | 19 | 10 | 69.25 | 189 | 51668 | 4790082 |
| MAP-517 | Sheep | 1889414 | 61 | 99 | 2548 | 1343 | 16 | 11 | 69.26 | 161 | 61823 | 4794394 |
| MAP-520 | Bison | 1390354 | 55 | 99 | 634 | 297 | - | - | 69.34 | 108 | 92217 | 4729308 |
| MAP-521 | Cattle | 1986976 | 65 | 99 | 143 | 59 | - | - | 69.32 | 129 | 74421 | 4750350 |
| MAP-524 | Cattle | 2111958 | 67 | 99 | 129 | 55 | - | - | 69.33 | 105 | 91963 | 4732046 |
| MAP-525 | Cattle | 1600582 | 53 | 99 | 98 | 37 | - | - | 69.33 | 120 | 86177 | 4742367 |
| MAP-526 | Cattle | 1526912 | 50 | 99 | 96 | 37 | - | - | 69.32 | 132 | 74421 | 4735896 |
| MAP-527 | Sheep | 2491406 | 79 | 99 | 3272 | 1517 | 16 | 8 | 69.27 | 153 | 65046 | 4801135 |
| MAP-529 | Cattle | 1597210 | 52 | 99 | 60 | 25 | - | - | 69.2 | 168 | 51514 | 4750955 |
| MAP-533 | Cattle | 1832792 | 63 | 99 | 130 | 56 | - | - | 69.33 | 111 | 85446 | 4751106 |
| MAP-534 | Cattle | 1683424 | 55 | 99 | 136 | 60 | - | - | 69.33 | 118 | 83179 | 4747022 |
| MAP-535 | Cattle | 1307336 | 43 | 99 | 135 | 59 | - | - | 69.33 | 114 | 91668 | 4748663 |
| MAP-536 | Cattle | 1109894 | 36 | 99 | 138 | 62 | - | - | 69.31 | 141 | 65011 | 4742575 |
| MAP-538 | Cattle | 1562410 | 54 | 99 | 144 | 59 | - | - | 69.32 | 128 | 80738 | 4753796 |
| MAP-539 | Cattle | 1445032 | 47 | 99 | 148 | 64 | - | - | 69.32 | 132 | 76999 | 4743348 |
| MAP-540 | Cattle | 1460488 | 50 | 99 | 140 | 58 | - | - | 69.33 | 115 | 83179 | 4748270 |
| MAP-541 | Cattle | 1449114 | 47 | 99 | 133 | 61 | - | - | 69.28 | 169 | 57762 | 4736631 |
| MAP-551 | Sheep | 1110326 | 35 | 99 | 3098 | 1507 | 12 | 137 | 69.26 | 172 | 56063 | 4788558 |
| MAP-552 | Sheep | 894506 | 29 | 99 | 3074 | 1501 | 253 | 138 | 69.18 | 253 | 37981 | 4745337 |
| MAP-554 | Cattle | 1791402 | 60 | 99 | 3353 | 1491 | 190 | 105 | 69.24 | 193 | 51332 | 4785668 |
| MAP-555 | Sheep | 1343068 | 44 | 99 | 142 | 60 | - | - | 69.32 | 136 | 66980 | 4743160 |
| MAP-556 | Sheep | 1646664 | 53 | 99 | 3313 | 1510 | 267 | 138 | 69.27 | 151 | 64932 | 4795795 |
| MAP-557 | Sheep | 1513596 | 49 | 99 | 3295 | 1511 | 168 | 138 | 69.28 | 141 | 80161 | 4798784 |
| MAP-558 | Sheep | 1314406 | 44 | 99 | 3345 | 1514 | 19 | 15 | 69.22 | 218 | 46438 | 4777007 |
| MAP-559 | Sheep | 969936 | 30 | 99 | 3030 | 1509 | 16 | 13 | 69.26 | 178 | 51219 | 4786991 |
| MAP-560 | Cattle | 1915412 | 63 | 99 | 138 | 64 | - | - | 69.32 | 124 | 86177 | 4749224 |
| MAP_561 | Sheep | 1038348 | 33 | 99 | 3018 | 1506 | 40 | 18 | 69.24 | 191 | 49192 | 4774918 |
| MAP-562 | Sheep | 1684650 | 53 | 99 | 3038 | 1513 | 36 | 18 | 69.28 | 147 | 61823 | 4799666 |
| MAP-564 | Cattle | 1641496 | 53 | 99 | 105 | 43 | - | - | 69.32 | 123 | 83523 | 4741667 |
| MAP-567 | Cattle | 1954034 | 65 | 99 | 102 | 44 | - | - | 69.29 | 149 | 59024 | 4730105 |
| MAP-568 | Cattle | 1620308 | 52 | 99 | 103 | 42 | - | - | 69.33 | 110 | 91896 | 4744427 |
| MAP-570 | Sheep | 1545306 | 49 | 99 | 3101 | 1507 | 39 | 18 | 69.29 | 135 | 83167 | 4805559 |
| MAP-571 | Sheep | 2348810 | 74 | 99 | 3014 | 1514 | 25 | 12 | 69.17 | 146 | 80747 | 4824058 |
| MAP-577 | Sheep | 1567846 | 50 | 99 | 3291 | 1507 | 41 | 20 | 69.2 | 143 | 69122 | 4822809 |
| MAP-579 | Sheep | 1786094 | 57 | 99 | 3320 | 1511 | 15 | 11 | 69.28 | 134 | 83167 | 4801777 |
| MAP-580 | Sheep | 1117780 | 35 | 99 | 3134 | 1503 | 38 | 16 | 69.29 | 131 | 80147 | 4806765 |
| MAP-581 | Sheep | 1834526 | 60 | 99 | 3332 | 1509 | 40 | 16 | 69.29 | 122 | 91573 | 4810172 |
| MAP-583 | Sheep | 1761868 | 54 | 99 | 3143 | 1504 | 41 | 17 | 69.28 | 133 | 83167 | 4799392 |
| MAP-584 | Sheep | 1514346 | 49 | 99 | 3287 | 1507 | 41 | 16 | 69.25 | 177 | 53513 | 4787965 |
| MAP-585 | Sheep | 2155156 | 71 | 99 | 3314 | 1506 | 43 | 19 | 69.28 | 144 | 73166 | 4803355 |
| MAP-586 | Sheep | 1851094 | 60 | 99 | 3341 | 1507 | 37 | 17 | 69.29 | 136 | 83167 | 4802234 |
| MAP-587 | Sheep | 1943282 | 63 | 99 | 3307 | 1506 | 40 | 20 | 69.19 | 147 | 65042 | 4816590 |
| MAP-588 | Sheep | 1014388 | 32 | 99 | 3060 | 1503 | 37 | 20 | 69.27 | 150 | 61823 | 4796743 |
| MAP-589 | Sheep | 1605356 | 50 | 99 | 3075 | 1508 | 39 | 20 | 69.2 | 133 | 84895 | 4821419 |
| MAP-592 | Sheep | 1051918 | 34 | 99 | 2972 | 1496 | 33 | 16 | 69.27 | 164 | 58790 | 4771474 |
| MAP-594 | Sheep | 1559564 | 47 | 99 | 3032 | 1509 | 11 | 7 | 69.28 | 132 | 80732 | 4802920 |
| MAP-595 | Cattle | 987270 | 30 | 99 | 83 | 38 | - | - | 69.32 | 122 | 86178 | 4738739 |
| MAP-596 | Cattle | 1001412 | 33 | 99 | 123 | 51 | - | - | 69.32 | 126 | 74421 | 4746250 |
| MAP-597 |  | 1091792 | 35 | 99 | 3283 | 1507 | 12 | 8 | 69.28 | 144 | 74539 | 4796629 |
| MAP-598 |  | 1741456 | 54 | 99 | 3138 | 1512 | 22 | 12 | 69.27 | 155 | 58777 | 4791312 |
| MAP-599 | Cattle | 1039328 | 33 | 99 | 135 | 61 | - | - | 69.32 | 121 | 86197 | 4747960 |
| MAP-602 | Sheep | 1212324 | 38 | 99 | 3238 | 1507 | 42 | 25 | 69.27 | 149 | 73636 | 4793421 |
| MAP-607 | Cattle | 2068276 | 68 | 99 | 135 | 59 | - | - | 69.34 | 108 | 91979 | 4753057 |
| MAP-608 | Cattle | 1820430 | 59 | 99 | 142 | 59 | - | - | 69.33 | 108 | 90142 | 4752521 |
| MAP-609 | Cattle | 2096986 | 71 | 99 | 72 | 30 | - | - | 69.31 | 131 | 76809 | 4746556 |
| MAP-610 | Cattle | 2553116 | 82 | 99 | 140 | 59 | - | - | 69.31 | 137 | 69211 | 4793176 |
| MAP-611 | Cattle | 2551304 | 85 | 99 | 142 | 60 | - | - | 69.33 | 113 | 83991 | 4747158 |
| MAP-612 | Cattle | 2091544 | 67 | 99 | 3339 | 1513 | 12 | 6 | 69.27 | 158 | 58775 | 4793176 |
| MAP-613 | Sheep | 1181284 | 37 | 99 | 3058 | 1500 | 36 | 18 | 69.21 | 222 | 42279 | 4757545 |
| MAP-617 | Sheep | 2250082 | 75 | 99 | 3277 | 1512 | 43 | 18 | 69.28 | 150 | 68511 | 4799069 |
| MAP-618 | Cattle | 2552520 | 81 | 99 | 129 | 58 | - | - | 69.31 | 131 | 65051 | 4747295 |
| MAP-619 | Cattle | 1830030 | 57 | 99 | 87 | 41 | - | - | 69.28 | 157 | 57782 | 4767064 |
| MAP-621 | Sheep | 1154684 | 34 | 99 | 2835 | 1503 | 13 | 9 | 69.16 | 207 | 46649 | 4799402 |
| MAP-623 | Sheep | 1244764 | 39 | 99 | 3033 | 1505 | 20 | 11 | 69.24 | 186 | 51273 | 4746132 |
| MAPMRI0102 | Cattle | 15287246 | 213 | 99 | 251 | 109 | - | - | 69.22 | 434 | 16886 | 4663840 |
| MAPMRI0104 | Cattle | 18187726 | 253 | 99 | 265 | 105 | - | - | 69.26 | 364 | 21312 | 4640503 |
| MAPMRI0106 | Cattle | 25749472 | 360 | 99 | 255 | 116 | - | - | 69.16 | 495 | 14609 | 4606503 |
| MAPMRI0107 | Cattle | 29215620 | 410 | 99 | 252 | 100 | - | - | 69.11 | 562 | 12813 | 4564073 |
| MAPMRI0108 | Cattle | 22116872 | 308 | 99 | 252 | 107 | - | - | 69 | 734 | 9746 | 4467654 |
| MAPMRI0109 | Cattle | 20013210 | 279 | 99 | 244 | 112 | - | - | 69.01 | 733 | 9426 | 4429452 |
| MAPMRI0111 | Cattle | 16793104 | 232 | 99 | 271 | 109 | - | - | 69.28 | 482 | 15363 | 4628201 |
| MAPMRI0112 | Cattle | 16556944 | 229 | 99 | 253 | 106 | - | - | 69.28 | 446 | 18934 | 4670248 |
| MAPMRI0113 | Cattle | 9762452 | 134 | 99 | 248 | 101 | - | - | 69.18 | 552 | 13173 | 4604777 |
| MAPMRI0114 | Cattle | 17817636 | 245 | 99 | 249 | 110 | - | - | 69.26 | 473 | 17382 | 4593859 |
| MAPMRI0115 | Cattle | 29290686 | 403 | 99 | 283 | 107 | - | - | 69.33 | 401 | 21663 | 4673028 |
| MAPMRI0116 | Cattle | 21820282 | 301 | 99 | 253 | 107 | - | - | 69.36 | 381 | 22465 | 4644826 |
| MAPMRI0117 | Bison | 20680660 | 285 | 99 | 687 | 283 | - | - | 69.32 | 411 | 21082 | 4668002 |
| MAPMRI0118 | Cattle | 25265054 | 351 | 99 | 263 | 113 | - | - | 69.24 | 481 | 14773 | 4581689 |
| MAPMRI0119 | Cattle | 11845778 | 168 | 99 | 257 | 98 | - | - | 69.08 | 570 | 13298 | 4594910 |
| MAPMRI0121 | Cattle | 14254858 | 203 | 99 | 238 | 94 | - | - | 69.03 | 656 | 11366 | 454236 |
| MAPMRI0122 | Cattle | 14054200 | 198 | 99 | 139 | 56 | - | - | 69.21 | 384 | 20508 | 4674372 |
| MAPMRI0123 | Cattle | 13272010 | 187 | 99 | 271 | 108 | - | - | 69.23 | 380 | 21240 | 4680768 |
| MAPMRI0124 | Cattle | 13058218 | 184 | 99 | 271 | 102 | - | - | 69.33 | 236 | 38253 | 4725570 |
| MAPMRI0125 | Cattle | 13325466 | 189 | 99 | 281 | 106 | - | - | 69.22 | 413 | 20400 | 4673529 |
| MAPMRI0126 | Cattle | 15118460 | 214 | 99 | 241 | 102 | - | - | 69.15 | 459 | 16477 | 4638520 |
| MAPMRI0127 | Bison | 12979520 | 184 | 99 | 658 | 283 | - | - | 69.24 | 359 | 22888 | 4689603 |
| MAPMRI0128 | Bison | 15863326 | 226 | 99 | 698 | 284 | - | - | 69.09 | 541 | 14000 | 4593394 |
| MAPMRI0129 | Cattle | 12751308 | 181 | 99 | 258 | 107 | - | - | 69.15 | 497 | 14770 | 4621561 |
| MAPMRI0131 | - | 69687800 | 1027 | 99 | 266 | 101 | - | - | 69.36 | 101 | 108014 | 4745187 |
| MAPMRI0132 | Cattle | 63109706 | 935 | 99 | 269 | 103 | - | - | 69.32 | 182 | 51874 | 4739599 |
| MAPMRI0133 | - | 32366146 | 478 | 99 | 269 | 104 | - | - | 69.31 | 177 | 56039 | 4734641 |
| MAPMRI0134 | Cattle | 45825100 | 680 | 99 | 46 | 20 | - | - | 69.24 | 267 | 36535 | 4698736 |
| MAPMRI0135 | - | 33510782 | 494 | 99 | 48 | 19 | - | - | 69.33 | 130 | 83178 | 4741142 |
| MAPMRI014 | Cattle | 2235506 | 31 | 98 | 132 | 49 | - | - | 68.95 | 602 | 11558 | 4505303 |
| MAPMRI022 | Cattle | 3106282 | 44 | 99 | 234 | 96 | - | - | 69.11 | 412 | 19269 | 4619360 |
| MAPMRI023 | Cattle | 2873702 | 41 | 99 | 145 | 55 | - | - | 69.15 | 372 | 20152 | 4639444 |
| MAPMRI026 | Cattle | 5213378 | 75 | 99 | 668 | 286 | - | - | 69.03 | 501 | 14577 | 4571001 |
| MAPMRI027 | Cattle | 3894732 | 53 | 99 | 253 | 112 | - | - | 69.15 | 373 | 20780 | 4645219 |
| MAPMRI028 | Cattle | 2891990 | 41 | 99 | 143 | 55 | - | - | 69.17 | 345 | 22129 | 4650277 |
| MAPMRI029 | Bison | 4146576 | 59 | 99 | 671 | 55 | - | - | 69.25 | 240 | 35955 | 4701707 |
| MAPMRI030 | Cattle | 3274456 | 47 | 99 | 216 | 77 | - | - | 69.27 | 196 | 39234 | 4693835 |
| MAPMRI031 | Bison | 2982326 | 42 | 99 | 675 | 292 | - | - | 69.26 | 242 | 37957 | 4699218 |
| MAPMRI032 | Cattle | 4030722 | 58 | 99 | 231 | 95 | - | - | 69.25 | 230 | 36548 | 4697908 |
| MAPMRI033 | Cattle | 2980430 | 43 | 99 | 241 | 100 | - | - | 69.24 | 250 | 34166 | 4677022 |
| MAPMRI034 | Bison | 6551264 | 96 | 99 | 689 | 293 | - | - | 69.23 | 263 | 34565 | 4698593 |
| MAPMRI036 | Cattle | 5908188 | 87 | 99 | 276 | 104 | - | - | 69.23 | 278 | 30555 | 4698561 |
| MAPMRI044 | Cattle | 3951072 | 49 | 99 | 110 | 47 | - | - | 69.22 | 246 | 35752 | 4690694 |
| MAPMRI049 | Sheep | 3189924 | 42 | 98 | 3425 | 1442 | 1671 | 753 | 68.99 | 524 | 14544 | 4606931 |
| MAPMRI050 | Cattle | 3634658 | 50 | 98 | 215 | 95 | - | - | 68.91 | 610 | 11052 | 4464705 |
| MAPMRI051 | Sheep | 2346766 | 32 | 98 | 3455 | 1464 | 1702 | 760 | 68.94 | 561 | 13073 | 4583646 |
| MAPMRI052 | Cattle | 2307914 | 30 | 98 | 234 | 101 | - | - | 68.69 | 816 | 7735 | 4309049 |
| MAPMRI053 | Cattle | 2238776 | 31 | 99 | 254 | 111 | - | - | 69.2 | 297 | 27890 | 4659517 |
| MAPMRI056 | Cattle | 2123234 | 30 | 99 | 223 | 96 | - | - | 69.15 | 356 | 23465 | 4626526 |
| MAPMRI058 | Sheep | 3249668 | 46 | 98 | 3487 | 1474 | 1704 | 780 | 69.18 | 274 | 31416 | 4728701 |
| MAPMRI059 | Cattle | 2140046 | 31 | 99 | 288 | 119 | - | - | 69.15 | 380 | 20323 | 4635042 |
| MAPMRI060 | - | 16246828 | 237 | 99 | 225 | 101 | - | - | 69.23 | 340 | 23404 | 4695827 |
| MAPMRI061 | - | 7773080 | 113 | 99 | 223 | 98 | - | - | 69.05 | 586 | 12991 | 4561798 |
| MAPMRI062 | - | 10642080 | 154 | 99 | 269 | 109 | - | - | 69.14 | 481 | 15712 | 4628361 |
| MAPMRI063 | - | 9335810 | 136 | 99 | 229 | 98 | - | - | 69.13 | 546 | 13916 | 4615787 |
| MAPMRI064 | - | 7085486 | 102 | 99 | 124 | 45 | - | - | 69.21 | 373 | 21208 | 4685941 |
| MAPMRI065 | Cattle | 13255726 | 193 | 99 | 98 | 48 | - | - | 69.33 | 159 | 50819 | 4741669 |
| MAPMRI066 | Cattle | 7697826 | 112 | 99 | 255 | 108 | - | - | 69.2 | 430 | 18461 | 4678937 |
| MAPMRI067 | Cattle | 22301198 | 326 | 99 | 252 | 106 | - | - | 69.26 | 290 | 35383 | 4706027 |
| MAPMRI068 | Cattle | 11407644 | 165 | 99 | 242 | 106 | - | - | 69.27 | 263 | 34675 | 4719168 |
| MAPMRI069 | Cattle | 8828708 | 129 | 99 | 145 | 55 | - | - | 69.17 | 449 | 17734 | 4652879 |
| MAPMRI070 | Cattle | 8567932 | 127 | 99 | 259 | 102 | - | - | 69.18 | 444 | 17230 | 4613195 |
| MAPMRI071 | Cattle | 12095338 | 178 | 99 | 114 | 51 | - | - | 69.27 | 266 | 31906 | 4693280 |
| MAPMRI072 | Cattle | 24611758 | 347 | 99 | 248 | 100 | - | - | 69.05 | 646 | 10893 | 4529663 |
| MAPMRI073 | Cattle | 26877532 | 383 | 99 | 230 | 104 | - | - | 68.87 | 833 | 7757 | 4357187 |
| MAPMRI074 | Sheep | 15915506 | 226 | 99 | 1033 | 440 | 3414 | 1441 | 68.97 | 753 | 9066 | 4437216 |
| MAPMRI075 | Cattle | 14617388 | 207 | 99 | 312 | 123 | - | - | 68.96 | 741 | 9162 | 4464586 |
| MAPMRI076 | Cattle | 12766720 | 181 | 99 | 237 | 92 | - | - | 69.05 | 645 | 10755 | 4531171 |
| MAPMRI077 | Cattle | 13542522 | 190 | 99 | 117 | 59 | - | - | 69.14 | 574 | 13607 | 4555674 |
| MAPMRI078 | Cattle | 18865316 | 266 | 99 | 233 | 103 | - | - | 69.13 | 566 | 12866 | 4553016 |
| MAPMRI080 | Cattle | 15138166 | 212 | 99 | 254 | 108 | - | - | 69.14 | 543 | 13053 | 4559047 |
| MAPMRI081 | Cattle | 16386328 | 231 | 99 | 231 | 95 | - | - | 69.08 | 632 | 11564 | 4577625 |
| MAPMRI082 | Cattle | 18529482 | 262 | 99 | 205 | 75 | - | - | 68.94 | 804 | 8750 | 4436830 |
| MAPMRI083 | Sheep | 22022468 | 305 | 99 | 1660 | 739 | 1660 | 739 | 69.03 | 652 | 10902 | 4555961 |
| MAPMRI084 | Cattle | 20704428 | 291 | 99 | 131 | 62 | - | - | 69.03 | 699 | 10480 | 4516056 |
| MAPMRI085 | Cattle | 6690456 | 93 | 98 | 3585 | 1463 | 1815 | 801 | 68.85 | 838 | 7979 | 4432098 |
| MAPMRI087 | Cattle | 15397642 | 215 | 99 | 281 | 108 | - | - | 69.14 | 519 | 14396 | 4613054 |
| MAPMRI088 | Cattle | 13280546 | 187 | 99 | 236 | 101 | - | - | 69.17 | 557 | 13802 | 4604717 |
| MAPMRI089 | Cattle | 16706978 | 234 | 99 | 219 | 91 | - | - | 69.14 | 583 | 13345 | 4583434 |
| MAPMRI091 | Cattle | 16640186 | 234 | 99 | 277 | 106 | - | - | 69.2 | 486 | 15927 | 4608274 |
| MAPMRI090 | Cattle | 15297916 | 214 | 99 | 231 | 100 | - | - | 69.16 | 509 | 15029 | 4602119 |
| MAPMRI094 | Sheep | 22490602 | 261 | 99 | 8434 | 88 | - | - | 68.9 | 594 | 14995 | 4984050 |
| MAPMRI095 | Cattle | 19612260 | 277 | 99 | 115 | 48 | - | - | 69 | 673 | 9980 | 4485483 |
| MAPMRI096 | Cattle | 12691196 | 179 | 99 | 223 | 98 | - | - | 68.91 | 788 | 8373 | 4422706 |
| MAPMRI097 | Cattle | 17664718 | 245 | 99 | 101 | 41 | - | - | 69.02 | 663 | 10534 | 4488736 |
| MAPMRI098 | Cattle | 15215278 | 213 | 99 | 114 | 46 | - | - | 69.11 | 571 | 12831 | 4554443 |
| MAPMRI099 | Cattle | 14508668 | 203 | 99 | 237 | 96 | - | - | 69.19 | 487 | 14884 | 4612819 |
| MAPMRI100 | Cattle | 13963512 | 195 | 99 | 220 | 98 | - | - | 69.16 | 501 | 15718 | 4611062 |
| MAPMRI101 | Cattle | 16246274 | 227 | 99 | 238 | 97 | - | - | 69.2 | 445 | 16059 | 4604956 |
| MAPMRI0103 | Cattle | 23743242 | 324 | 99 | 3586 | 1479 | 411 | 177 | 69.25 | 392 | 20494 | 4718080 |
| MAPMRI155 | Cattle | 1807644 | 52 | 99 | 48 | 17 | - | - | 69.21 | 244 | 38387 | 4691196 |
| MAPMRI156 | Cattle | 1192140 | 35 | 99 | 257 | 98 | - | - | 69.22 | 241 | 34995 | 4686130 |
| MAPMRI157 | Cattle | 1419284 | 42 | 99 | 268 | 103 | - | - | 69.3 | 157 | 58011 | 4732490 |
| MAPMRI158 | Cattle | 1475690 | 42 | 99 | 76 | 34 | - | - | 69.27 | 184 | 52621 | 4720974 |
| MAPMRI159 | Cattle | 1543406 | 45 | 99 | 98 | 37 | - | - | 69.35 | 93 | 99087 | 4756467 |
| MAPMRI160 | Cattle | 1470772 | 44 | 99 | 255 | 111 | - | - | 69.12 | 381 | 21259 | 4631276 |
| MAPMRI035 | Sheep | 5010586 | 70 | 99 | 235 | 103 | - | - | 69.2 | 312 | 27755 | 4678292 |
| SRR1793679 | Cattle | 1030612 | 35 | 99 | 136 | 56 | - | - | 69.36 | 96 | 117910 | 4764436 |
| SRR1793681 | Cattle | 1280614 | 47 | 99 | 116 | 51 | - | - | 69.35 | 80 | 125338 | 4764489 |
| SRR1793683 | Cattle | 1914758 | 70 | 99 | 107 | 43 | - | - | 69.35 | 80 | 125338 | 4763306 |
| SRR1793685 | Cattle | 2092900 | 83 | 99 | 684 | 290 | - | - | 69.36 | 78 | 128436 | 4768669 |
| SRR1793698 | Cattle | 1469854 | 46 | 99 | 125 | 53 | - | - | 69.35 | 80 | 130987 | 4764341 |
| SRR1793700 | Cattle | 1466088 | 75 | 99 | 150 | 65 | - | - | 69.36 | 85 | 125338 | 4762471 |
| SRR1793701 | Cattle | 1596098 | 51 | 99 | 113 | 45 | - | - | 69.36 | 83 | 125338 | 4727011 |
| SRR1793702 | Cattle | 1426140 | 44 | 99 | 109 | 44 | - | - | 69.35 | 84 | 125338 | 4761583 |
| SRR1793704 | Cattle | 1267394 | 34 | 99 | 96 | 66 | - | - | 69.35 | 87 | 117910 | 4754689 |
| SRR1793703 | Cattle | 1727556 | 55 | 99 | 148 | 40 | - | - | 69.36 | 79 | 125338 | 4769796 |
| SRR1793706 | Cattle | 1771234 | 52 | 99 | 114 | 47 | - | - | 69.35 | 79 | 125338 | 4761630 |
| SRR1793707 | Cattle | 2457572 | 81 | 99 | 248 | 98 | - | - | 69.36 | 75 | 125299 | 4774828 |
| SRR1793708 | Cattle | 1047252 | 33 | 99 | 101 | 36 | - | - | 69.35 | 81 | 125338 | 4752923 |
| SRR1793709 | Cattle | 1769676 | 55 | 99 | 242 | 102 | - | - | 69.36 | 82 | 125338 | 4762780 |
| SRR1793716 | Cattle | 1751732 | 57 | 99 | 221 | 99 | - | - | 69.35 | 77 | 125338 | 4761901 |
| SRR1793718 | Cattle | 1494128 | 47 | 99 | 142 | 65 | - | - | 69.36 | 84 | 125338 | 4771905 |
| SRR1793719 | Cattle | 1876720 | 61 | 99 | 125 | 54 | - | - | 69.36 | 75 | 125338 | 4768658 |
| SRR1793720 | Cattle | 1747662 | 57 | 99 | 78 | 26 | - | - | 69.36 | 84 | 117910 | 4760997 |
| SRR1793723 | Cattle | 1492656 | 48 | 99 | 112 | 46 | - | - | 69.35 | 76 | 125338 | 4762425 |
| SRR1793726 | Cattle | 1498082 | 46 | 99 | 105 | 42 | - | - | 69.36 | 81 | 125338 | 4767064 |
| DT3 | Cattle | 1987184 | 100 | 99 | 1728 | 683 | - | - | 69.33 | 78 | 130943 | 4767808 |
| E93 | Cattle | 2000000 | 99 | 99 | 1802 | 678 | - | - | 69.31 | 1 | 4786065 | 4786065 |
| MAP-4 | Cattle | 2000000 | 99 | 99 | 1782 | 704 | - | - | 69.3 | 1 | 4829424 | 4829424 |
| Tn-India | Bison | 2000000 | 99 | 99 | 2233 | 866 | - | - | 69.3 | 1 | 4829781 | 4829781 |
| E1 | Cattle | 2000000 | 100 | 99 | 2531 | 968 | - | - | 69.3 | 1 | 4781002 | 4781002 |
| CLIJ644-CSIRO | Cattle | 1986392 | 315 | 99 | 43057 | 644 | - | - | 69.33 | 78 | 130943 | 4767808 |

*’-‘ Indicates that SNPs were not analysed for Type C strains against Map Telford strain and Type S strains against Map k10
